# Exercise capacity in heart failure: a systematic review and meta-analysis of HFrEF and HFpEF disparities in VO_2_peak and 6-minute walking distance

**DOI:** 10.1093/ehjopen/oeaf055

**Published:** 2025-05-14

**Authors:** Konstantinos Prokopidis, Krzysztof Irlik, Theocharis Ispoglou, Panagiotis Ferentinos, Alexandros Mitropoulos, Mathias Schlögl, Masoud Isanejad, Kamil Kegler, Katarzyna Nabrdalik, Gregory Y H Lip

**Affiliations:** Department of Musculoskeletal Ageing and Science, Institute of Life Course and Medical Sciences, University of Liverpool, 6 West Derby St, Liverpool L7 8TX, UK; Liverpool Centre for Cardiovascular Science at University of Liverpool, Liverpool John Moores University and Liverpool Heart & Chest Hospital, Thomas Dr, Liverpool L14 3PE, UK; Students' Scientific Association by the Department of Internal Medicine, Diabetology and Nephrology in Zabrze, Faculty of Medical Sciences in Zabrze, Medical University of Silesia, Katowice, Poland; Doctoral School, Department of Internal Medicine, Diabetology and Nephrology, Faculty of Medical Sciences in Zabrze, Medical University of Silesia, Poniatowskiego 15, 40-055 Katowice, Poland; Carnegie School of Sport, Leeds Beckett University, Leeds LS1 3HE, UK; Carnegie School of Sport, Leeds Beckett University, Leeds LS1 3HE, UK; Department of Nursing and Midwifery, Sheffield Hallam University, Howard St, Sheffield City Centre, Sheffield S1 1WB, UK; University Clinic for Acute Geriatric Care, City Hospital Waid Zurich, Tièchestrasse 99, 8037 Zürich, Switzerland; Department of Musculoskeletal Ageing and Science, Institute of Life Course and Medical Sciences, University of Liverpool, 6 West Derby St, Liverpool L7 8TX, UK; Students' Scientific Association by the Department of Internal Medicine, Diabetology and Nephrology in Zabrze, Faculty of Medical Sciences in Zabrze, Medical University of Silesia, Katowice, Poland; Liverpool Centre for Cardiovascular Science at University of Liverpool, Liverpool John Moores University and Liverpool Heart & Chest Hospital, Thomas Dr, Liverpool L14 3PE, UK; Department of Internal Medicine, Diabetology and Nephrology, Faculty of Medical Sciences in Zabrze, Medical University of Silesia, Poniatowskiego 15, 40-055 Katowice, Poland; Liverpool Centre for Cardiovascular Science at University of Liverpool, Liverpool John Moores University and Liverpool Heart & Chest Hospital, Thomas Dr, Liverpool L14 3PE, UK; Department of Clinical Medicine, Danish Center for Health Services Research, Aalborg University, Fredrik Bajers Vej 7K, 9220 Aalborg Øst, Aalborg, Denmark

**Keywords:** Heart failure, VO_2peak_, Physical function, HFrEF, HFpEF

## Abstract

**Aims:**

Heart failure (HF) with reduced ejection fraction (HFrEF) and HF with preserved ejection fraction (HFpEF) exhibit unique physiological pathways, influencing exercise capacity and functional performance. This systematic review and meta-analysis aimed to compare peak oxygen consumption (VO_2peak_), six-minute walk distance (6MWD), cardiac output (CO), and stroke volume (SV), between these phenotypes.

**Methods and results:**

A systematic literature search of cohort studies via databases (PubMed, Web of Science, Scopus, and Cochrane Library) was conducted from inception until October 2024. A meta-analysis using a random-effects model to calculate the pooled effects was employed. Forty-six studies were included. HFrEF patients demonstrated significantly greater 6MWD compared to HFpEF (*k* = 20; mean difference (MD): 18.09 m, 95% confidence interval (CI) 1.59–34.59, I^2^ = 86%, *P* = 0.03), though this difference became insignificant after adjusting for comorbidities. Conversely, HFpEF patients exhibited higher VO_2peak_ (*k* = 20; MD: −0.78 mL/kg/min, 95% CI −1.45–−0.11, I^2^ = 89%, *P* = 0.02), CO (*k* = 12; MD: −1.15 L/min, 95% CI −2.11–−0.19, I^2^ = 97%, *P* = 0.02), and SV (*k* = 14; SMD: −1.00, 95% CI −1.60–−0.39, I^2^ = 95%, *P* < 0.01). Age was identified as a significant moderator of VO_2peak_.

**Conclusion:**

HFpEF patients demonstrated superior VO_2peak_, CO, and SV compared to HFrEF patients, while the observed 6MWD advantage in HFrEF was likely influenced by comorbidities. Our findings emphasize the importance of tailoring rehabilitation strategies to HF phenotype-specific physiological profiles, particularly focusing on improving VO_2peak_ and cardiac efficiency in HFpEF.

## Introduction

Heart failure (HF) is major clinical and public health problem designated as an emerging epidemic since 1997.^1^ This is condition consists of two distinct phenotypes identified based on ejection fraction (EF).^2^ These phenotypes are HF with reduced ejection fraction (HFrEF) and HF with preserved ejection fraction (HFpEF) sharing common pathophysiological pathways,^3,4^ but exhibiting distinct physiological profiles that impact exercise capacity and functional performance beyond EF alone. Heart failure is categorized into four stages based on the relationship between symptoms of dyspnoea and physical activity using The New York Heart Association (NYHA) classification.

In patients with mild HF (NYHA II), cardiac output (CO) may appear normal at rest but fails to increase with physical activity.^5^ The relationship between maximal oxygen consumption (VO_2peak)_, peak CO, and muscle perfusion suggests that inadequate CO increase may trigger anaerobic metabolism at lower workloads, contributing to muscle fatigue. Consequently, patients with HF often reach a symptom-limited VO_2_, commonly referred to as ‘VO_2peak_’, instead of a true VO_2peak_, further highlighting the need for careful interpretation of these values in clinical practice.

Assessment of patient’s exertional capacity may support exercise prescription and provide insights into HF severity^6^ and this can be performed with the measurement of VO_2peak_, reflecting the cardiopulmonary system’s capacity during exercise.^7^ Previous research indicates that lower VO_2peak_ in HFrEF is linked to reduced stroke volume (SV) and CO due to impaired systolic function.^8^ VO_2peak_, often measured via symptom limited cardiopulmonary exercise (CPET) is a valuable prognostic tool for both HFrEF and HFpEF.^9,10^

CPET, involving gas analysis during progressive exercise, assesses minute ventilation (Ve), oxygen uptake (O_2_), and carbon dioxide (CO_2_),^10^ helps to identify maladaptive physiological responses to exercise and combined with other metrics such as heart rate, blood pressure, and electrocardiogram, enables personalized exercise prescriptions, enhancing clinical insights into exercise intolerance.^11^

Although a symptom limited CPET is an objective measure of functional capacity, the six-minute walk distance test (6MWD) offers a simpler measure to assess functional capacity and endurance in this population,^12^ particularly for those with advanced diseases and multiple comorbidities^13^ (Giannitsi *et al*. 2019). Including 6MWD data alongside VO_2peak_ and CPET measures may provide a more comprehensive understanding of patient exercise tolerance, supporting the development of more effective exercise interventions.

Considering that exercise can positively affect VO_2peak_ in both HF phenotypes,^14^ understanding potential differences in VO_2peak_, 6MWD, SV, and CO may be pivotal for optimizing exercise prescriptions and rehabilitation strategies appropriate to each phenotype.

The primary aim of this systematic review and meta-analysis was to systematically compare VO_2peak_, 6MWD, and related parameters in HFrEF and HFpEF, addressing current knowledge gaps and providing evidence to guide more effective interventions for each phenotype. These may clarify how various end criteria in VO_2_ testing, such as those discussed in Edvardsen *et al.* (2014), may impact VO_2_ measurements, helping to inform adjustments in exercise protocols based on patient characteristics such as age and sex.^15^

## Methods

The revised 2020 Preferred Reporting Items for Systematic Reviews and Meta-Analyses criteria were followed,^16^ with a protocol registered in the International Prospective Register of Systematic Reviews (PROSPERO) (CRD42024495582).

### Inclusion and exclusion criteria

#### Inclusion

- Data pertaining to HFrEF (mean LVEF ≤ 40%) and HFpEF (mean LVEF ≥ 50%) with mean age 18 years of age or above.
- Data collection will only be eligible from studies that include information for both HF phenotypes.
- Studies may be interventional or observational.

#### Exclusion

- Data collection from HFrEF (mean LVEF > 40 and ≤ 50%) and HFpEF (mean LVEF 40–49%).
- Not published in English.

### Search strategy

From inception to October 2024, four databases (PubMed, Cochrane Library, Scopus, and Web of Science) were independently searched by two investigators. A detailed description of the keyword search strategy is displayed in Supplementary material online, *Table S1*.

### Outcomes of interest

We gathered data related to VO_2peak_, 6MWD, SV, and CO from both HFrEF and HFpEF based on VO_2peak_ was measured in mL/kg/min, CO in L/min, SV in mL, and 6MWD in meters (m).

### Data extraction and risk of bias

Two investigators extracted data independently, including details such as the name of the first author, country of origin, participant age, sex, and body mass index (BMI), study design, LVEF rate, phenotype of HF, definition of HF phenotype, VO_2peak_ method of assessment, brain natriuretic peptide levels, reported comorbidities, and outcomes of interest. Any disagreements were resolved by a third investigator. The Newcastle–Ottawa scale (NOS) was utilized to assess study quality/risk of bias (RoB) for cohort studies. NOS assigns a maximum of nine points across three quality parameters: Selection, comparability, and outcome. The evaluation was made by two investigators, and it was classified as high (≤5 points), moderate (6–7 points), or low (8–9 points). For cross-sectional studies, it was classified as high (≤3 points, moderate (4–5 points), or low (6–7 points).^17^ For randomized controlled trials (RCTs), the quality of the studies was evaluated using the risk-of-bias 2 (RoB2) tool.^18^ RoB2 assesses bias according to five domains: (i) randomization process; (ii) deviations from intended interventions; (iii) missing outcome data; (iv) measurement of the outcome; and (v) selection of the reported result. Based on its scoring system, bias was defined as ‘high’, ‘some concerns’, or ‘low’.

### Statistical analysis

Quantitative data were considered as continuous measurements, and differences in outcomes between those with HFrEF vs. HFpEF were compared to determine MDs or standardized MDs (SMDs) in case units of assessment were not uniform. Statistical heterogeneity of outcome measurements across studies was measured using the overlap of their confidence intervals (CI 95%) and expressed as Cochran’s Q (χ^2^ test) and I^2^ measurements.^19^

The random-effects model and the inverse-variance approach were used to determine statistical significance set at *P* < 0.05. The meta-analysis was synthesized using Review Manager (RevMan 5.4.1) software. Furthermore, low heterogeneity was defined as I^2^ between 30% and 49%, moderate heterogeneity between 50% and 74%, and high heterogeneity at 75% and above.^20^ Sensitivity analysis was performed to assess the robustness of reported statistical results by controlling for studies with increased risk of bias. In the case of substantial heterogeneity, a random-effects meta-regression was carried out to investigate potential sources of variability that could alter estimate rates across studies.^21^ Particularly, meta-regressions included factors such as age and BMI. Potential publication bias was evaluated using funnel plots and Egger’s weighted regression test to quantitatively assess asymmetry in study results.^22^

## Results

**Figure 1:**
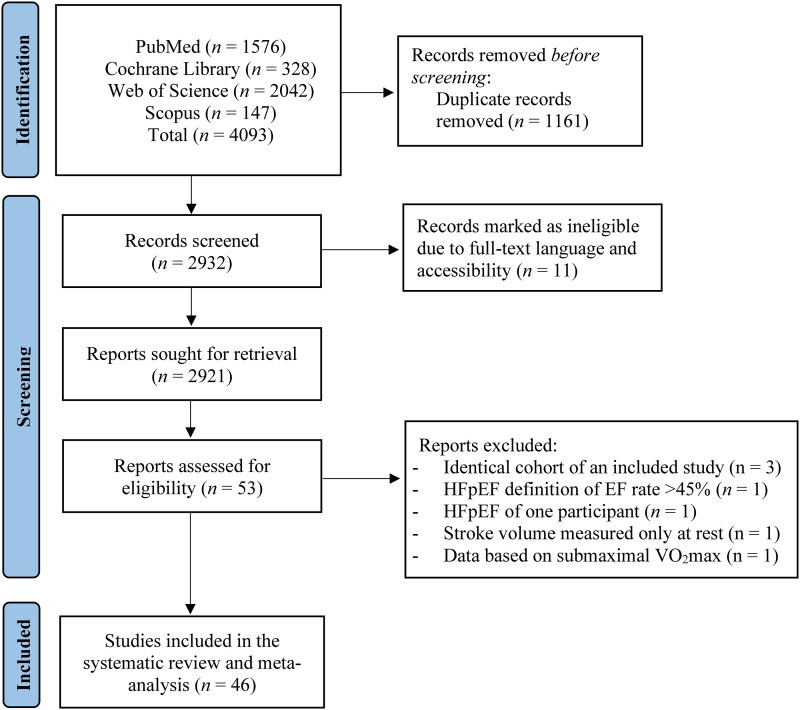
Literature search for the included studies.

The initial literature search provided 4093 publications. Following the exclusion of duplicates, abstracts, studies that full text could not be obtained and in a different language, 53 full texts were identified as potentially eligible for inclusion in the systematic review and meta-analysis. Of these 53 articles, three could not be included considering a more recent eligible cohort for inclusion in our study, one study defined HFpEF as EF rate above 45%, one study included only one HFpEF participant that could not be converted in our model, one study measured SV only at rest, while another study did not measure VO_2_ on maximal capacity. Overall, 46 studies were included in the systematic review and meta-analysis (Flowchart—*Figure 1*), for which, detailed characteristics are presented in *Table 1*.

**Table 1:** Study and participant characteristics of the included studies

Study	Total *n* (M/F)	HFrEF			HFpEF		
		*n* (M/F)	Age	BMI	*n* (M/F)	Age	BMI
Abe *et al*.^23^	23 (1/12)	13 (1/12)	75 ± 7.0	–	10 (7/3)	65 ± 12.0	–
Adams *et al*.^24^	40 (15/5)	20 (15/5)	60.1 ± 1.7	30.4 ± 1.4	20 (5/15)	69.7 ± 1.6	33 ± 1.4
Arvidsson *et al*.^25^	32 (−/-)	16 (−/−)	65.7 ± 8.1	–	16 (−/−)	70.3 ± 13.8	–
Bekfani *et al*.^26^	35 (15/3)	18 (15/3)	68 ± 9.0	–	17 (8/9)	71 ± 6.0	28.7 ± 4.6
Blum *et al.*^27^	35 (15/3)	18 (15/3)	65.4 ± 10.5	28.1 ± 3.8	17 (9/8)	77.9 ± 8.0	27.6 ± 3.8
Charman *et al.*^28^	38 (−/−)	21 (−/−)	–	–	17 (−/−)	–	–
Chung *et al.*^29^	40 (17/3)	20 (17/3)	64 ± 10.0	28.3 ± 5.8	20 (14/6)	64 ± 8.0	30.2 ± 5.5
Conti *et al*.^30^	47 (16/8)	24 (16/8)	63.5 ± 9.6	28.1 ± 4.73	23 (13/10)	63.9 ± 10.3	27.9 ± 2.8
Daubert *et al.* (OMT alone)^31^	37 (−/−)	28 (−/−)	62.6 ± 12.7	–	9 (−/−)	68.2 ± 12.7	–
de Denus *et al.*^32^	54 (−/−)	28 (−/−)	63 ± 12.6	–	26 (−/−)	75.6 ± 11.9	–
Dhakal *et al.*^33^	104 (45/11)	56 (45/11)	59 ± 12.0	27.8 ± 6.0	48 (20/28)	63 ± 12.0	33.7 ± 7.6
Edlund *et al.*^34^	30 (12/3)	15 (12/3)	66 ± 8.2	–	15 (11/4)	71 ± 18.0	–
Fudim *et al.*^35^	441 (145/80)	225 (145/80)	62.7 ± 11.2	29.6 ± 6.0	216 (112/104)	69.3 ± 11.2	33.4 ± 8.0
Fujiwara *et al.*^36^	143 (38/6)	44 (38/6)	58 ± 14.0	23.4 ± 4.4	99 (81/18)	67 ± 12.0	24.2 ± 2.9
Gong *et al.*^37^	1183 (430/168)	598 (430/168)	58.3 ± 13.1	28.2 ± 5.4	585 (287/298)	58.1 ± 16.0	29.9 ± 7.1
Guazzi *et al.*^38^	68 (26/8)	34 (26/8)	63 ± 9.0	–	34 (26/8)	62.7 ± 9.3	–
Hou *et al.*^39^	37 (14/3)	17 (14/3)	–	–	20 (11/9)	–	–
Hsu *et al.* (GDMT group)^40^	99 (57/16)	73 (57/16)	57.8 ± 3.6	25.5 ± 1.3	26 (16/10)	65.3 ± 5.3	26.4 ± 2.6
Hsu *et al.* (HIIT group)^40^	79 (51/14)	65 (51/14)	59.7 ± 4.9	25.5 ± 1.7	14 (8/6)	66.2 ± 10.7	26.3 ± 2.88
Hundley *et al.*^41^	17 (4/4)	8 (4/4)	73 ± 7.0	27 ± 5.0	9 (3/6)	74 ± 7.0	30 ± 10.0
Ingle *et al.*^42^	672 (430/138)	568 (430/138)	74.3 ± 8.2	26.8 ± 4.5	104 (57/47)	75.3 ± 9.8	30.4 ± 5.3
Kanagala *et al.*^43^	186 (23/23)	46 (23/23)	72 ± 8.0	28 ± 6.0	140 (68/72)	73 ± 9.0	34 ± 7.0
Li *et al.*^44^	89 (19/29)	48 (19/29)	71.6 ± 4.5	28.4 ± 2.3	41 (26/15)	65.1 ± 4.3	28.6 ± 2.3
Luo *et al.*^45^	84 (31/2)	33 (31/2)	56.5 ± 15.3	25.6 ± 5.5	51 (42/9)	62.3 ± 6.0	25.4 ± 2.8
Maldonado-Martin *et al.*^46^	97 (33/17)	50 (33/17)	69.4 ± 5.2	26.7 ± 4.3	47 (6/41)	68.8 ± 6.1	30.5 ± 6.0
Moriwaki *et al.*^47^	20 (7/3)	10 (7/3)	53 ± 11.0	25 ± 4.0	10 (2/8)	68 ± 18.0	24 ± 6.0
Namasivayam *et al.*^48^	203 (34/13)	57 (34/13)	60 ± 13.0	27.4 ± 4.4	146 (74/72)	63 ± 13.0	32.9 ± 7.5
Obokata *et al.*^49^	80 (28/15)	43 (28/15)	67 ± 13.0	21 ± 2.8	37 (22/15)	70 ± 11.0	22.2 ± 3.3
Paolisso *et al.*^50^	56 (24/11)	35 (24/11)	67 ± 13.0	27 ± 5.0	21 (5/16)	75 ± 9.0	30 ± 7.0
Pugliese *et al.*^51^	99 (42/12)	54 (42/12)	63.9 ± 11.3	25.7 ± 3.4	45 (32/13)	64.3 ± 12.1	26.9 ± 4.7
Rickenbacher *et al.*^52^	514 (271/131)	402 (271/131)	75.5 ± 7.5	25.3 ± 4.1	112 (40/72)	80.2 ± 7.1	27 ± 5.4
Sato *et al.*^53^	6 (−/−)	3 (−/−)	50.7 ± 20.5	–	3 (−/−)	60.3 ± 10.0	–
Sato *et al.*^54^	936 (419/79)	498 (419/79)	59.1 ± 14.4	22.9 ± 4.1	438 (339/99)	61.8 ± 14.3	23.8 ± 4.1
Schwartzenberg *et al.*^55^	257 (149/25)	174 (149/25)	56 ± 12.0	29.5 ± 5.8	83 (12/71)	69 ± 9.0	33.2 ± 8.3
Scrutinio *et al.*^56^	1547 (951/217)	1168 (951/217)	65.3 ± 12.3	–	379 (176/203)	73.6 ± 11.9	–
Shah *et al.*^57^	317 (86/11)	97 (86/11)	74.5 ± 7.3	–	220 (197/23)	74.7 ± 7.1	–
Steding-Ehrenborg *et al.*^58^	21 (6/4)	10 (6/4)	66 ± 14.6	27 ± 4.8	11 (4/7)	72 ± 15.3	28.8 ± 3.2
Steding-Ehrenborg *et al.*^59^	30 (12/3)	15 (12/3)	63.3 ± 18.8	29.4 ± 14.1	15 (8/7)	70.7 ± 21.3	29.3 ± 14.1
Sugimoto *et al.*^60^	147 (79/26)	105 (79/26)	65.5 ± 12.3	26.3 ± 3.9	42 (19/23)	70.8 ± 10.2	28.4 ± 5.0
Vale-Lira *et al.*^61^	28 (11/1)	12 (11/1)	54.4 ± 7.3	28.3 ± 5.1	16 (8/8)	55.6 ± 11.5	30 ± 4.0
Van Iterson *et al.*^62^	59 (30/2)	32 (30/2)	55 ± 10.0	28 ± 4.0	27 (16/11)	71 ± 11.0	33 ± 6.0
Vuckovic *et al.*^63^	45 (14/12)	26 (14/12)	64.3 ± 1.6	33.9 ± 1.6	19 (7/12)	65.7 ± 2.4	33.7 ± 1.8
Wang *et al.*^64^	209 (25/11)	36 (25/11)	68.3 ± 12.6	24.1 ± 4.8	173 (80/93)	71.8 ± 11.8	24.6 ± 3.8
Warraich *et al.*^65^	202 (56/50)	106 (56/50)	72.3 ± 7.7	30.6 ± 7.5	96 (37/59)	71.7 ± 7.4	36.1 ± 9.3
Wernhart *et al.*^66^	276 (130/23)	153 (130/23)	52.8 ± 10.3	28.2 ± 4.6	123 (72/51)	60 ± 12.3	27.2 ± 5.2
Wisniacki *et al.*^67^	52 (16/11)	27 (16/11)	79.8 ± 5.2	23.2 ± 2.7	25 (12/13)	80.4 ± 4.5	25.5 ± 3.6
Zile *et al.*^68^	929 (376/155)	531 (376/155)	67.2 ± 11.4	31.4 ± 7.4	398 (200/198)	71.6 ± 9.7	36.3 ± 9.0

Data are expressed as mean ± standard deviation.

As detailed in Supplementary material online, *Table S2*, VO₂_peak_ was assessed via treadmill protocols in two studies and cycle ergometry in the remaining 44 studies. Left ventricular EF was predominantly measured by echocardiography with some studies using cardiac magnetic resonance imaging (cMRI), ventriculography, or employing mixed methods. The age of participants ranged from 50.7 to 80.4 years, with BMI values ranging from 21 to 36.1 kg/m^2^. On average, participants with HFpEF were older and had higher BMI compared to those with HFrEF.

### Heart failure with reduced ejection fraction vs. heart failure with preserved ejection fraction: six-minute walk distance

**Figure 2:**
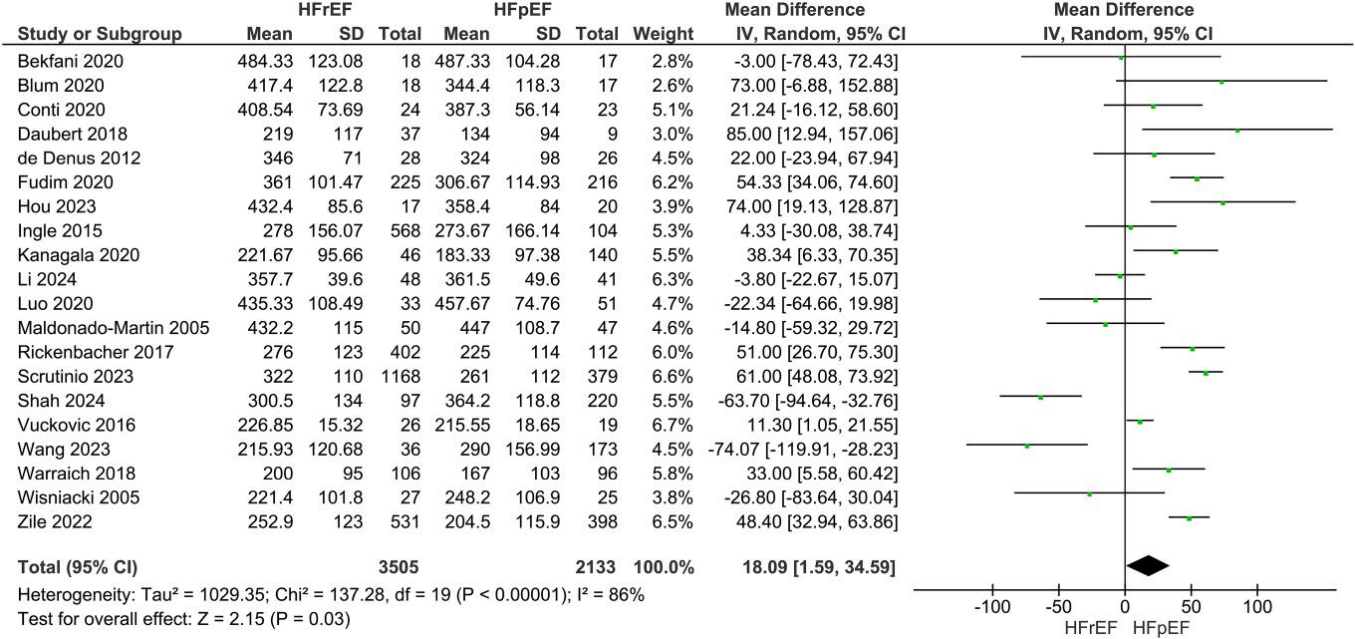
Differences in six-minute walking distance between heart failure with reduced ejection fraction (HFrEF) and heart failure with preserved ejection fraction (HFpEF).

Our main analysis showed significantly greater 6MWD in HFrEF vs. HFpEF (*k* = 20; MD: 18.09 m, 95% CI 1.59–34.59, I^2^ = 86%, *P* = 0.03) (*Figure 2*). When studies, where a phenotype had higher reported comorbidities over the other, were excluded the findings became statistically insignificant (*k* = 13; MD: 10.97 m, 95% CI −10.87–32.32, I^2^ = 87%, *P* = 0.32) (see Supplementary material online, *Figure S1*). Exclusion of a study with high risk of bias did not alter the findings of the main analysis (*k* = 19; MD: 19.86 m, 95% CI 3.09–36.63, I^2^ = 87%, *P* = 0.02) (see Supplementary material online, *Figure S2*).

### Heart failure with reduced ejection fraction vs. heart failure with preserved ejection fraction: VO_2peak_, cardiac output, and stroke volume

**Figure 3:**
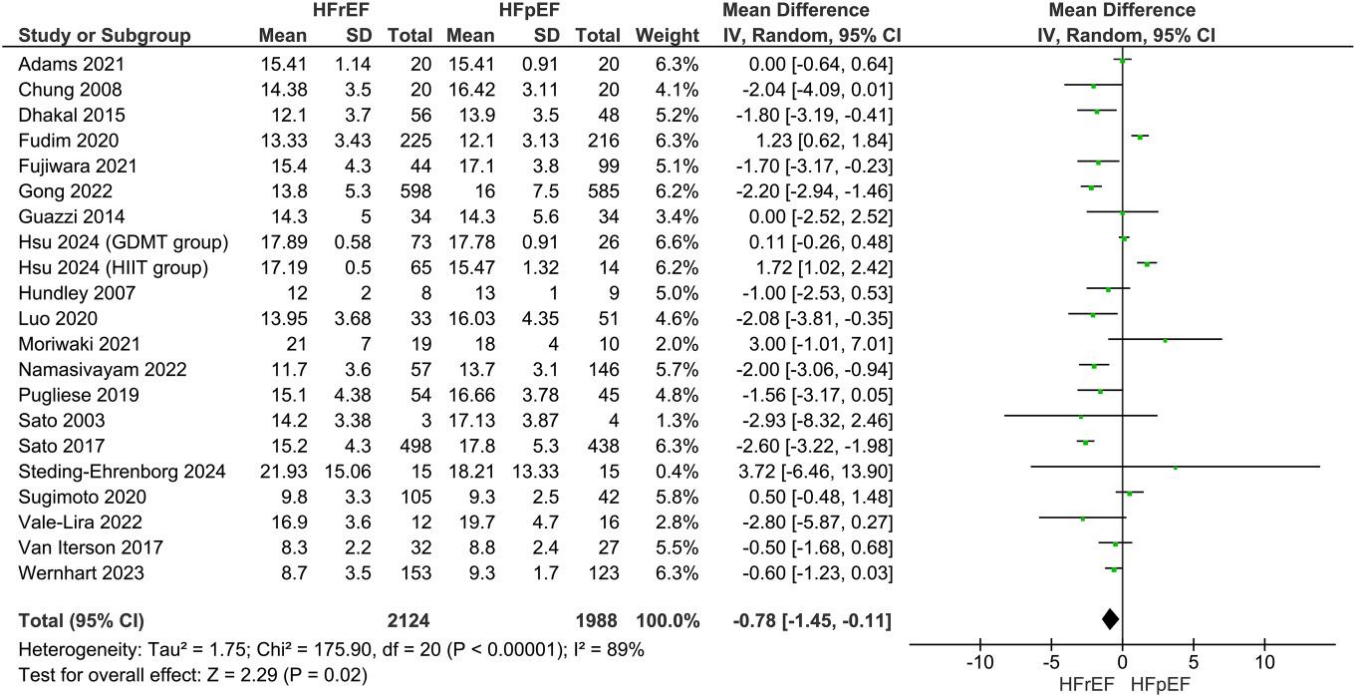
Differences in VO_2peak_ between heart failure with reduced ejection fraction (HFrEF) and heart failure with preserved ejection fraction (HFpEF).

**Figure 4:**
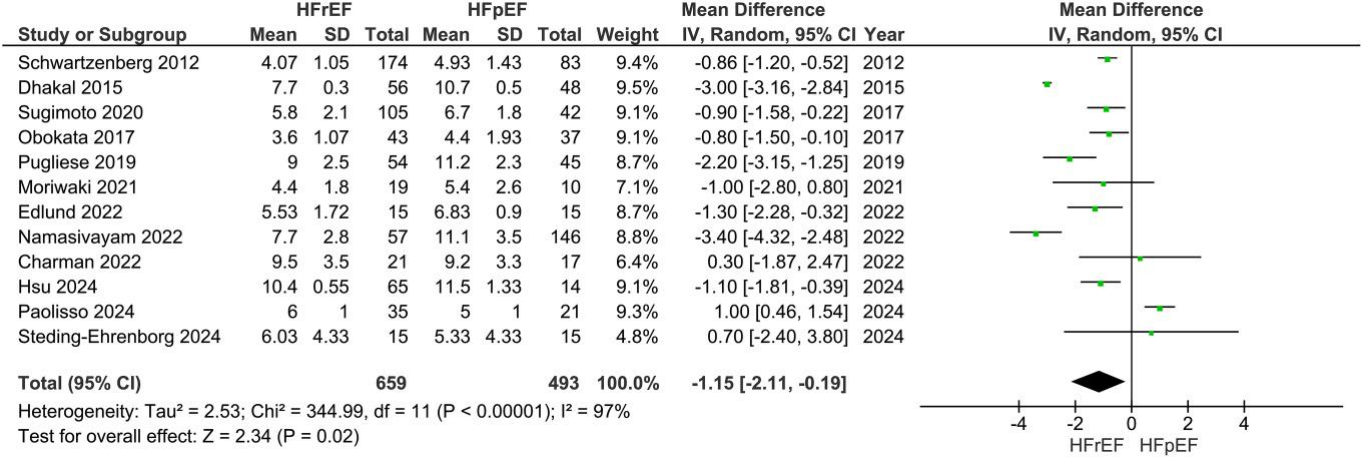
Differences in cardiac output between heart failure with reduced ejection fraction (HFrEF) and heart failure with preserved ejection fraction (HFpEF).

**Figure 5:**
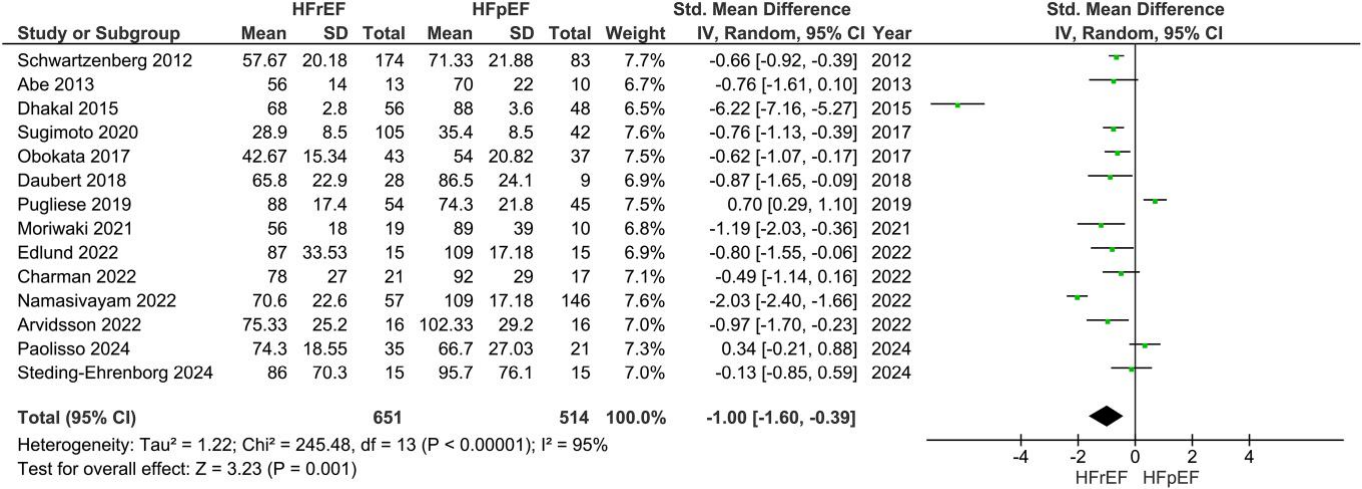
Differences in stroke volume between heart failure with reduced ejection fraction (HFrEF) and heart failure with preserved ejection fraction (HFpEF).

Our main analysis showed significantly lower VO_2peak_ in HFrEF vs. HFpEF (*k* = 20; MD: −0.78 mL/kg/min, 95% CI −1.45–−0.11, I^2^ = 89%, *P* = 0.02) (*Figure 3*). However, sensitivity analysis excluding studies where one phenotype had higher reported comorbidities over the other rendered the results statistically insignificant (*k* = 15; MD: −0.70 mL/kg/min, 95% CI −1.60–0.19, I^2^ = 91%, *P* = 0.12) (see Supplementary material online, *Figure S3*). Sensitivity analyses excluding studies with high risk of bias also revealed insignificant findings (*k* = 16; −0.77 mL/kg/min, 95% CI −1.56–0.02, I^2^ = 91%, *P* = 0.06) (see Supplementary material online, *Figure S4*). In addition, these results were accompanied by statistically significant decreases in CO (*k* = 12; MD: −1.15 L/min, 95% CI −2.11–−0.19, I^2^ = 97%, *P* = 0.02) (*Figure 4*) and SV (*k* = 14; SMD: −1.00, 95% CI −1.60–−0.39, I^2^ = 95%, *P* < 0.01) (*Figure 5*). To measure the MD of SV differences between phenotypes, a sensitivity analysis was conducted to remove a study that used SV index. This analysis confirmed that HFrEF had significantly lower SV (in mL) compared to HFpEF (*k* = 13; MD: −15.05 mL, 95% CI −22.92–−7.18, I^2^ = 91%, *P* < 0.01) (see Supplementary material online, *Figure S5*). Sensitivity analyses excluding studies with additional reported comorbidities did not alter the results of the main analysis for either CO (*k* = 9; MD: −1.23 L/min, 95% CI −2.19–−0.28, I^2^ = 93%, *P* = 0.01) (see Supplementary material online, *Figure S6*) or SV (*k* = 9; −14.99 mL, 95% CI −25.98–−4.00, I^2^ = 89%, *P* < 0.01) (see Supplementary material online, *Figure S7*).

### Publication bias and meta-regression

Potential publication bias based on CO was found (*P* = 0.0314), indicating statistically significant funnel plot asymmetry (see Supplementary material online, *Table S3*; Supplementary material online, Supplementary file; CO—Publication bias). No publication bias was detected for VO_2peak_, SV, and 6MWD (see Supplementary material online, *Table S3*). Meta-regression analysis demonstrated that age was a significant moderator of VO_2peak_ (*z* = 2.19, *P* = 0.03, 95% CI 0.02–0.28). In contrast, the proportion of females and BMI were not significant moderators of VO_2peak_ in this meta-analysis (*P* > 0.05; Supplementary material online, *Table S4*).

### Risk of bias assessment

No studies with high risk of bias were found regarding the included RCTs. One study had some concerns, particularly in relation to some missing data, for which, methods to handle them were not fully described, lack of clarity in the blinding of some outcomes, and no specific method of sequence generation described (see Supplementary material online, *Table S5*). Furthermore, risk of bias assessment of the included cohort studies showed no studies with increased risk (see Supplementary material online, *Table S6*), however, six cross-sectional studies were evaluated as having an increased bias risk (see Supplementary material online, *Table S7*).

## Discussion

This systematic review and meta-analysis revealed that patients with HFrEF exhibited greater 6MWD compared to HFpEF, however, those with HFpEF demonstrated a greater VO_2peak_, SV, and CO. These disparities highlight the distinct pathophysiological profiles of these phenotypes and reflect differences in age, comorbidities, and cardiovascular function that influence exercise capacity.

### Walking tests and cardiac economy

Our findings on 6MWD disparities between HFrEF and HFpEF complement existing research on walking tests as functional metrics in cardiac populations. However, the clinical significance of 6MWD differences may be limited without incorporating measures of cardiac efficiency. For example, the Heart Rate Walking Speed Index (HRWSI), which evaluates heart rate in relation to walking speed, provides additional insights into cardiac economy and exercise-induced adaptations.^69^ This metric could complement traditional walking tests, helping to distinguish between true physiological improvements and variability caused by external factors.

Pertinent to 6MWD, exclusion of studies where one phenotype had an increasing number of reported comorbidities over the other led to a 10.97 m difference favouring HFrEF. This modest difference, although statistically significant in the main analysis, raises questions about its clinical relevance. The 6MWD test is subject to a learning effect, as demonstrated by previous research in patients with asthma and patients with chronic HF, where test-retest reliability testing resulted to improvements of up to 35 m.^70^ Our findings align with these observations, suggesting that the small differences in 6MWD between phenotypes may reflect test variability rather than meaningful functional disparities. Despite this, 6MWD remains a valuable metric for functional capacity, particularly in settings where CPET is not feasible.

In patients with asthma, there was a mean increase of 18 m (95% CI 11to 24 m) in 6MWD (73% of the sample showed improvement),^70^ demonstrating a learning effect that could explain such differences. This number is identical to our findings of 18.09 m (95% CI 1.59–34.59 m). In addition, in patients with chronic HF, learning effect was even greater (31 m (95% CI 27–35 m) during a second attempt,^71^ therefore, given that the included studies did not report whether another attempt was made and the between-test differences, 6MWD changes between phenotypes may have negligible clinical value.

### Oxygen capacity and VO_2_peak

Peak VO₂ is a validated prognostic marker in HF, though its role in evaluating functional capacity changes post-intervention remains debated.^72,73^ Although the main analysis showed statistically significant differences in VO_2peak_ favouring HFpEF, sensitivity analyses accounting for comorbidities and study quality rendered these findings non-significant. VO_2peak_ differences of −0.78 mL/kg/min (95% CI −1.45–−0.11 mL/kg/min) (main analysis) or −0.70 mL/kg/min (95% CI −1.60–0.19 mL/kg/min) (comorbidities-adjusted analysis) align with prior research suggesting that even small changes in VO_2peak_ (i.e. 6% change or 1 mL/kg/min) can have prognostic value,^74^ however, such outcome was based on repeat test variability rather than clinical significance. Nevertheless, in patients with chronic HF, every 6% increase in VO_2peak_ is linked to an 8% lower risk of all-cause mortality and a 7% lower risk of cardiovascular mortality or? HF hospitalization.^72^

Interestingly, our meta-regression identified age as a significant moderator of VO_2peak_, reiterating the importance of specifically designing interventions to address age-related declines in oxygen capacity. Despite these nuances, VO_2peak_ appears to offer greater prognostic value in HFpEF compared to HFrEF. Prior research supports this, with VO_2peak_ strongly linked to clinical outcomes in HFpEF.^75^ Accordingly, exercise interventions that target oxygen capacity, such as 3–12 months of structured exercise training, could confer meaningful benefits in terms of VO_2peak_, exercise duration, and quality of life,^76^ while reducing and HF-related hospitalizations.^77^ These findings align with the observed associations between VO_2peak_, SV, and CO, which were significantly higher in HFpEF compared to HFrEF.

### Fidelity of exercise programs

Another important consideration in understanding exercise outcomes is the fidelity of exercise delivery. As demonstrated in cardiac rehabilitation studies, adherence to prescribed exercise intensities and durations (e.g. > 50% HRR for ≥20 min) significantly influences improvements in cardiorespiratory fitness.^69^ Inconsistent fidelity across exercise training studies may partially explain the variability observed in VO_2peak_ and 6MWD outcomes observed in this review. While our study identified differences between HFrEF and HFpEF, these findings are potentially impacted by inconsistencies in intervention intensity and monitoring across studies. Ensuring rigorous fidelity monitoring in future research could improve the reliability and replicability of findings from exercise interventions.

### Physiological mechanisms and phenotype-specific differences

These observed differences in exercise capacity and oxygen dynamics may be explained by distinct physiological perturbations underpinning each phenotype. For example, patients with HFrEF experience more severe reductions in pulmonary oxygen uptake kinetics during exercise, deoxy-haemoglobin kinetics, and microvascular oxygen delivery compared with HFpEF.^78^ In addition, a recent meta-analyses identified low left ventricular global longitudinal strain as a stronger determinant of decreased VO_2peak_, a parameter more prevalent in HFrEF.^79^ Mitochondrial dysfunction is another critical factor with HFrEF associated with reduced energy supply due to lower levels of complex I, malate dehydrogenase, and creatine kinase activity.^24,80^ These differences provide a mechanistic basis for poorer exercise capacity and cardiac function in HFrEF.

### Strengths and limitations

In this study, consistent rates of LVEF were employed to categorize HFrEF and HFpEF ensuring robust comparisons between phenotypes. Sources of heterogeneity, including age and BMI, were explored through meta-regression, offering insights into moderating factors. However, these results are based on cross-sectional data, which do not imply causative implications between the two HF phenotypes. Although almost every study utilized cycle ergometry for VO₂_peak_ assessment instead of treadmill, variability in exercise protocols (e.g. ramp vs. stepwise increments) may introduce heterogeneity in VO₂_peak_ measurements. Additionally, left ventricular EF assessment predominantly relied on echocardiography, though some studies used alternative methods (cMRI, ventriculography, or mixed modalities), and cross-validation between methods was rarely reported. Additionally, publication bias was observed for CO, suggesting caution in interpreting these findings. Lastly, comorbidities and medication count among studies may have been over- or underreported due to potential inaccuracies arising from errors in drug prescription coding or incorrect electronic tabulations.

## Conclusion

This systematic review and meta-analysis highlights distinct differences between HFrEF and HFpEF in terms of exercise capacity and oxygen dynamics. While HFrEF patients demonstrated superior 6MWD, HFpEF patients exhibited higher VO_2peak_, SV, and CO. These findings highlight the nuanced nature of exercise capacity disparities between phenotypes, influenced by factors such as age and comorbidities. Further research should focus on longitudinal studies to track changes in VO_2peak_, 6MWD, and HRWSI over time and explore phenotype-specific rehabilitation strategies. Optimizing exercise interventions that target oxygen capacity and cardiac efficiency holds significant promise for improving clinical outcomes in both HFrEF and HFpEF populations.

## Supplementary Material

oeaf055_Supplementary_Data

## Data Availability

Data is available upon request.

## References

[1] Braunwald E . Cardiovascular medicine at the turn of the millennium: triumphs, concerns, and opportunities. N Engl J Med 1997;337:1360–1369. 9358131 10.1056/NEJM199711063371906

[2] McDonagh TA, Metra M, Adamo M, , Gardner RS, Baumbach A, Böhm M, Burri H, Butler J, Čelutkienė J, Chioncel O, Cleland JGF, Coats AJS, Crespo-Leiro MG, Farmakis D, Gilard M, Heymans S, Hoes AW, Jaarsma T, Jankowska EA, Lainscak M, Lam CSP, Lyon AR, McMurray JJV, Mebazaa A, Mindham R, Muneretto C, Piepoli MF, Price S, Rosano GMC, Ruschitzka F, Skibelund AK; ESC Scientific Document Group. 2021 ESC guidelines for the diagnosis and treatment of acute and chronic heart failure: developed by the task force for the diagnosis and treatment of acute and chronic heart failure of the European Society of Cardiology (ESC) with the special contribution of the Heart Failure Association (HFA) of the ESC. Rev Esp Cardiol (Engl Ed). 2022;75:523. 35636830 10.1016/j.rec.2022.05.005

[3] Jin X, Nauta JF, Hung C-L, Ouwerkerk W, Teng T-HK, Voors AA, Lam CS, van Melle JP. Left atrial structure and function in heart failure with reduced (HFrEF) versus preserved ejection fraction (HFpEF): systematic review and meta-analysis. Heart Fail Rev 2022;27:1933–1955. 35079942 10.1007/s10741-021-10204-8 PMC9388424

[4] Simmonds SJ, Cuijpers I, Heymans S, Jones EA. Cellular and molecular differences between HFpEF and HFrEF: a step ahead in an improved pathological understanding. Cells 2020;9:242. 31963679 10.3390/cells9010242 PMC7016826

[5] Del Buono MG, Arena R, Borlaug BA, Carbone S, Canada JM, Kirkman DL, Garten R, Rodriguez-Miguelez P, Guazzi M, Lavie CJ, Abbate A. Exercise intolerance in patients with heart failure: JACC state-of-the-art review. J Am Coll Cardiol 2019;73:2209–2225. 31047010 10.1016/j.jacc.2019.01.072

[6] Balady GJ, Arena R, Sietsema K, Myers J, Coke L, Fletcher GF, Forman D, Franklin B, Guazzi M, Gulati M, Keteyian SJ, Lavie CJ, Macko R, Mancini D, Milani RV. Clinician’s guide to cardiopulmonary exercise testing in adults: a scientific statement from the American Heart Association. Circulation 2010;122:191–225. 20585013 10.1161/CIR.0b013e3181e52e69

[7] Hawkins MN, Raven PB, Snell PG, Stray-Gundersen J, Levine BD. Maximal oxygen uptake as a parametric measure of cardiorespiratory capacity. Med Sci Sports Exerc 2007;39:103–107. 17218891 10.1249/01.mss.0000241641.75101.64

[8] Bailey CS, Wooster LT, Buswell M, Patel S, Pappagianopoulos PP, Bakken K, White C, Tanguay M, Blodgett JB, Baggish AL, Malhotra R, Lewis GD. Post-exercise oxygen uptake recovery delay: a novel index of impaired cardiac reserve capacity in heart failure. JACC Heart Fail 2018;6:329–339. 29525330 10.1016/j.jchf.2018.01.007 PMC5880321

[9] Shafiq A, Brawner CA, Aldred HA, Lewis B, Williams CT, Tita C, Schairer JR, Ehrman JK, Velez M, Selektor Y, Lanfear DE, Keteyian SJ. Prognostic value of cardiopulmonary exercise testing in heart failure with preserved ejection fraction. The henry ford HospITal CardioPulmonary EXercise testing (FIT-CPX) project. Am Heart J 2016;174:167–172. 26995385 10.1016/j.ahj.2015.12.020 PMC4804356

[10] Malhotra R, Bakken K, D’Elia E, Lewis GD. Cardiopulmonary exercise testing in heart failure. JACC Heart Fail 2016;4:607–616. 27289406 10.1016/j.jchf.2016.03.022

[11] Arena R, Myers J, Guazzi M. Cardiopulmonary exercise testing is a core assessment for patients with heart failure. Congest Heart Fail 2011;17:115–119. 21609384 10.1111/j.1751-7133.2011.00216.x

[12] Giannitsi S, Bougiakli M, Bechlioulis A, Kotsia A, Michalis LK, Naka KK. 6-minute walking test: a useful tool in the management of heart failure patients. Ther Adv Cardiovasc Dis 2019;13:1753944719870084. 31441375 10.1177/1753944719870084 PMC6710700

[13] Guazzi M, Dickstein K, Vicenzi M, Arena R. Six-minute walk test and cardiopulmonary exercise testing in patients with chronic heart failure: a comparative analysis on clinical and prognostic insights. Circ Heart Fail 2009;2:549–555. 19919979 10.1161/CIRCHEARTFAILURE.109.881326

[14] Tucker WJ, Lijauco CC, Hearon CM Jr, Angadi SS, Nelson MD, Sarma S, Nanayakkara S, La Gerche A, Haykowsky MJ. Mechanisms of the improvement in peak VO2 with exercise training in heart failure with reduced or preserved ejection fraction. Heart Lung Circ 2018;27:9–21. 28870770 10.1016/j.hlc.2017.07.002

[15] Edvardsen E, Hem E, Anderssen SA. End criteria for reaching maximal oxygen uptake must be strict and adjusted to sex and age: a cross-sectional study. PLoS One 2014;9:e85276. 24454832 10.1371/journal.pone.0085276 PMC3891752

[16] Page MJ, Moher D, Bossuyt PM, Boutron I, Hoffmann TC, Mulrow CD, Shamseer L, Tetzlaff JM, Akl EA, Brennan SE, Chou R, Glanville J, Grimshaw JM, Hróbjartsson A, Lalu MM, Li T, Loder EW, Mayo-Wilson E, McDonald S, McGuinness LA, Stewart LA, Thomas J, Tricco AC, Welch VA, Whiting P, McKenzie JE. PRISMA 2020 explanation and elaboration: updated guidance and exemplars for reporting systematic reviews. BMJ 2021;372:n160. 33781993 10.1136/bmj.n160 PMC8005925

[17] Luchini C, Stubbs B, Solmi M, Veronese N. Assessing the quality of studies in meta-analyses: advantages and limitations of the Newcastle Ottawa scale. World J Metaanal 2017;5:80–84.

[18] Sterne JA, Savović J, Page MJ, Elbers RG, Blencowe NS, Boutron I, Cates CJ, Cheng H-Y, Corbett MS, Eldridge SM, Emberson JR, Hernán MA, Hopewell S, Hróbjartsson A, Junqueira DR, Jüni P, Kirkham JJ, Lasserson T, Li T, McAleenan A, Reeves BC, Shepperd S, Shrier I, Stewart LA, Tilling K, White IR, Whiting PF, Higgins JPT. Rob 2: a revised tool for assessing risk of bias in randomised trials. BMJ 2019;366:l4898. 31462531 10.1136/bmj.l4898

[19] Hozo SP, Djulbegovic B, Hozo I. Estimating the mean and variance from the median, range, and the size of a sample. BMC Med Res Methodol 2005;5:13. 15840177 10.1186/1471-2288-5-13 PMC1097734

[20] Higgins JP, Thompson SG, Deeks JJ, Altman DG. Measuring inconsistency in meta-analyses. BMJ 2003;327:557–560. 12958120 10.1136/bmj.327.7414.557 PMC192859

[21] Cumpston M, Li T, Page MJ, Chandler J, Welch VA, Higgins JP, Thomas J. Updated guidance for trusted systematic reviews: a new edition of the cochrane handbook for systematic reviews of interventions. Cochrane Database Syst Rev 2019;10:ED000142. 31643080 10.1002/14651858.ED000142 PMC10284251

[22] Lin L, Chu H. Quantifying publication bias in meta-analysis. Biometrics 2018;74:785–794. 29141096 10.1111/biom.12817 PMC5953768

[23] Abe H, Caracciolo G, Kheradvar A, Pedrizzetti G, Khandheria BK, Narula J, Sengupta PP. Contrast echocardiography for assessing left ventricular vortex strength in heart failure: a prospective cohort study. Eur Heart J Cardiovasc Imaging 2013;14:1049–1060. 23588788 10.1093/ehjci/jet049

[24] Adams V, Wunderlich S, Mangner N, Hommel J, Esefeld K, Gielen S, Halle M, Ellingsen Ø, Van Craenenbroeck EM, Wisløff U, Pieske B, Linke A, Winzer EB. Ubiquitin-proteasome-system and enzymes of energy metabolism in skeletal muscle of patients with HFpEF and HFrEF. ESC Heart Fail 2021;8:2556–2568. 33955206 10.1002/ehf2.13405 PMC8318515

[25] Arvidsson M, Ahmed A, Bouzina H, Rådegran G. Plasma proteoglycan prolargin in diagnosis and differentiation of pulmonary arterial hypertension. ESC Heart Fail 2021;8:1230–1243. 33403810 10.1002/ehf2.13184 PMC8006732

[26] Bekfani T, Bekhite Elsaied M, Derlien S, Nisser J, Westermann M, Nietzsche S, Hamadanchi A, Fröb E, Westphal J, Haase D, Kretzschmar T, Schlattmann P, Smolenski UC, Lichtenauer M, Wernly B, Jirak P, Lehmann G, Möbius-Winkler S, Schulze PC. Skeletal muscle function, structure, and metabolism in patients with heart failure with reduced ejection fraction and heart failure with preserved ejection fraction. Circ Heart Fail 2020;13:e007198. 33302709 10.1161/CIRCHEARTFAILURE.120.007198

[27] Blum M, Hashemi D, Motzkus LA, Neye M, Dordevic A, Zieschang V, Zamani SM, Lapinskas T, Runte K, Kelm M, Kühne T, Tahirovic E, Edelmann F, Pieske B, Düngen H-D, Kelle S. Variability of myocardial strain during isometric exercise in subjects with and without heart failure. Front Cardiovasc Med 2020;7:111. 32714945 10.3389/fcvm.2020.00111 PMC7344153

[28] Charman SJ, Okwose NC, Taylor CJ, Bailey K, Fuat A, Ristic A, Mant J, Deaton C, Seferovic PM, Coats AJS, Hobbs FDR, MacGowan GA, Jakovljevic DG. Feasibility of the cardiac output response to stress test in suspected heart failure patients. Fam Pract 2022;39:805–812. 35083480 10.1093/fampra/cmab184 PMC9508869

[29] Chung I, Goyal D, Macfadyen RJ, Lip GYH. The effects of maximal treadmill graded exercise testing on haemorheological, haemodynamic and flow cytometry platelet markers in patients with systolic or diastolic heart failure. Eur J Clin Invest 2008;38:150–158. 18257777 10.1111/j.1365-2362.2008.01909.x

[30] Conti V, Corbi G, Polito MV, Ciccarelli M, Manzo V, Torsiello M, De Bellis E, D'Auria F, Vitulano G, Piscione F, Carrizzo A, Di Pietro P, Vecchione C, Ferrara N, Filippelli A. Sirt1 activity in PBMCs as a biomarker of different heart failure phenotypes. Biomolecules 2020;10:1590. 33238655 10.3390/biom10111590 PMC7700185

[31] Daubert MA, Whellan DJ, Woehrle H, Tasissa G, Anstrom KJ, Lindenfeld J, Benjafield A, Blase A, Punjabi N, Fiuzat M, Oldenburg O, O'Connor CM. Treatment of sleep-disordered breathing in heart failure impacts cardiac remodeling: insights from the CAT-HF trial. Am Heart J 2018;201:40–48. 29910054 10.1016/j.ahj.2018.03.026

[32] de Denus S, Lavoie J, Ducharme A, O'Meara E, Racine N, Sirois MG, Neagoe P-E, Zhu L, Rouleau J-L, White M. Differences in biomarkers in patients with heart failure with a reduced vs a preserved left ventricular ejection fraction. Can J Cardiol 2012;28:62–68. 22104539 10.1016/j.cjca.2011.09.007

[33] Dhakal BP, Malhotra R, Murphy RM, Pappagianopoulos PP, Baggish AL, Weiner RB, Houstis NE, Eisman AS, Hough SS, Lewis GD. Mechanisms of exercise intolerance in heart failure with preserved ejection fraction: the role of abnormal peripheral oxygen extraction. Circ Heart Fail 2015;8:286–294. 25344549 10.1161/CIRCHEARTFAILURE.114.001825 PMC5771713

[34] Edlund J, Arvidsson PM, Nelsson A, Smith JG, Magnusson M, Heiberg E, Steding-Ehrenborg K, Arheden H. Noninvasive assessment of left ventricular pressure-volume relations: inter-and intraobserver variability and assessment across heart failure subtypes. Am J Cardiol 2022;184:48–55. 36192197 10.1016/j.amjcard.2022.09.001

[35] Fudim M, Kelly JP, Jones AD, AbouEzzeddine OF, Ambrosy AP, Greene SJ, Reddy YNV, Anstrom KJ, Alhanti B, Lewis GD, Hernandez AF, Felker GM. Are existing and emerging biomarkers associated with cardiorespiratory fitness in patients with chronic heart failure? Am Heart J 2020;220:97–107. 31805424 10.1016/j.ahj.2019.11.006 PMC7008085

[36] Fujiwara K, Shimada K, Nishitani-Yokoyama M, Kunimoto M, Matsubara T, Matsumori R, Abulimiti A, Aikawa T, Ouchi S, Shimizu M, Fukao K, Miyazaki T, Honzawa A, Yamada M, Saitoh M, Morisawa T, Takahashi T, Daida H, Minamino T. Arterial stiffness Index and exercise tolerance in patients undergoing cardiac rehabilitation comparison between patients with preserved and reduced ejection fraction. Int Heart J 2021;62:230–237. 33731517 10.1536/ihj.20-418

[37] Gong J, Castro RR, Caron JP, Bay CP, Hainer J, Opotowsky AR, Mehra MR, Maron BA, Di Carli MF, Groarke JD, Nohria A. Usefulness of ventilatory inefficiency in predicting prognosis across the heart failure spectrum. ESC Heart Fail 2022;9:293–302. 34931762 10.1002/ehf2.13761 PMC8788025

[38] Guazzi M, Labate V, Cahalin LP, Arena R. Cardiopulmonary exercise testing reflects similar pathophysiology and disease severity in heart failure patients with reduced and preserved ejection fraction. Eur J Prev Cardiol 2014;21:847–854. 23382540 10.1177/2047487313476962

[39] Hou X, Hashemi D, Erley J, Neye M, Bucius P, Tanacli R, Kühne T, Kelm M, Motzkus L, Blum M, Edelmann F, Kuebler WM, Pieske B, Düngen H-D, Schuster A, Stoiber L, Kelle S. Noninvasive evaluation of pulmonary artery stiffness in heart failure patients via cardiovascular magnetic resonance. Sci Rep 2023;13:22656. 38114509 10.1038/s41598-023-49325-5 PMC10730605

[40] Hsu CC, Fu TC, Wang CH, Huang TS, Cherng WJ, Wang JS. High-intensity interval training is associated with improved 10-year survival by mediating left ventricular remodeling in patients with heart failure with reduced and mid-range ejection fraction. J Am Heart Assoc 2024;13:e031162. 38240219 10.1161/JAHA.123.031162 PMC11056167

[41] Hundley WG, Bayram E, Hamilton CA, Hamilton EA, Morgan TM, Darty SN, Stewart KP, Link KM, Herrington DM, Kitzman DW. Leg flow-mediated arterial dilation in elderly patients with heart failure and normal left ventricular ejection fraction. Am J Physiol Heart Circ Physiol 2007;292:H1427–H1434. 17085542 10.1152/ajpheart.00567.2006

[42] Ingle L, Cleland JG, Clark AL. Perception of symptoms is out of proportion to cardiac pathology in patients with “diastolic heart failure”. Heart 2008;94:748–753. 18070942 10.1136/hrt.2007.131144

[43] Kanagala P, Arnold JR, Singh A, Chan DCS, Cheng ASH, Khan JN, Gulsin GS, Yang J, Zhao L, Gupta P, Squire IB, Ng LL, McCann GP. Characterizing heart failure with preserved and reduced ejection fraction: an imaging and plasma biomarker approach. PLoS One 2020;15:e0232280. 32349122 10.1371/journal.pone.0232280 PMC7190371

[44] Li Z, Shi Y, Xia Y, Wu L, Li H, Zhou R, Gao X, Zhang H, Jin X, Zhang J. Disparate clinical characteristics and prognosis of HFpEF versus HFrEF phenotype of diabetic cardiomyopathy. J Clin Med 2023;12:1565. 36836101 10.3390/jcm12041565 PMC9960597

[45] Luo Q, Li C, Zhuang B, Li G, Luo L, Ni Y, Huang Z, Wang L, Song H, Yan W, Shen Y. Establishment of exercise intensity for patients with chronic heart failure equivalent to anaerobic threshold based on 6-minute walking test. Ann Palliat Med 2020;9:2766–2775. 32921092 10.21037/apm-20-265

[46] Maldonado-Martin S, Brubaker PH, Kaminsky LA, Moore JB, Stewart KP, Kitzman DW. The relationship of a 6-min walk to VO (2 peak) and VT in older heart failure patients. Med Sci Sports Exerc 2006;38:1047–1053. 16775543 10.1249/01.mss.0000222830.41735.14

[47] Moriwaki K, Fujimoto N, Omori T, Miyahara S, Kameda I, Ishiyama M, Sugiura E, Nakamori S, Dohi K. Comparison of haemodynamic response to muscle reflex in heart failure with reduced vs. Preserved ejection fraction. ESC Heart Fail 2021;8:4882–4892. 34725954 10.1002/ehf2.13682 PMC8712776

[48] Namasivayam M, Lau ES, Zern EK, Schoenike MW, Hardin KM, Sbarbaro JA, Cunningham TF, Farrell RM, Rouvina J, Kowal A, Bhat RR, Brooks LC, Nayor M, Shah RV, Ho JE, Malhotra R, Lewis GD. Exercise blood pressure in heart failure with preserved and reduced ejection fraction. Heart Failure 2022;10:278–286. 35361448 10.1016/j.jchf.2022.01.012 PMC9730937

[49] Obokata M, Nagata Y, Kado Y, Kurabayashi M, Otsuji Y, Takeuchi M. Ventricular-arterial coupling and exercise-induced pulmonary hypertension during low-level exercise in heart failure with preserved or reduced ejection fraction. J Card Fail 2017;23:216–220. 27737768 10.1016/j.cardfail.2016.10.001

[50] Paolisso P, Gallinoro E, Belmonte M, Bertolone DT, Bermpeis K, De Colle C, Shumkova M, Leone A, Caglioni S, Esposito G, Fabbricatore D, Moya A, Delrue L, Penicka M, De Bruyne B, Barbato E, Bartunek J, Vanderheyden M. Coronary microvascular dysfunction in patients with heart failure: characterization of patterns in HFrEF versus HFpEF. Circ Heart Fail 2024;17:e010805. 38108151 10.1161/CIRCHEARTFAILURE.123.010805

[51] Pugliese NR, Fabiani I, Santini C, Rovai I, Pedrinelli R, Natali A, Dini FL. Value of combined cardiopulmonary and echocardiography stress test to characterize the haemodynamic and metabolic responses of patients with heart failure and mid-range ejection fraction. Eur Heart J Cardiovasc Imaging 2019;20:828–836. 30753369 10.1093/ehjci/jez014

[52] Rickenbacher P, Kaufmann BA, Maeder MT, Bernheim A, Goetschalckx K, Pfister O, Pfisterer M, Brunner-La Rocca H-P. Heart failure with mid-range ejection fraction: a distinct clinical entity? Insights from the trial of intensified versus standard medical therapy in elderly patients with congestive heart failure (TIME-CHF). Eur J Heart Fail 2017;19:1586–1596. 28295985 10.1002/ejhf.798

[53] Sato M, Maehara K, Yaoita H, Otani H, Hirosaka A, Saito T, Onuki N, Komatsu N, Ishihata T, Maruyama Y Correlation between cardiac norepinephrine overflow during exercise and cardiac 123I-MIBG uptake in patients with chronic heart failure. J Nucl Med 2003;44:1618–1624. 14530476

[54] Sato T, Yoshihisa A, Kanno Y, Suzuki S, Yamaki T, Sugimoto K, Kunii H, Nakazato K, Suzuki H, Saitoh S-I, Ishida T, Takeishi Y. Cardiopulmonary exercise testing as prognostic indicators: comparisons among heart failure patients with reduced, mid-range and preserved ejection fraction. Eur J Prev Cardiol 2017;24:1979–1987. 29086584 10.1177/2047487317739079

[55] Schwartzenberg S, Redfield MM, From AM, Sorajja P, Nishimura RA, Borlaug BA. Effects of vasodilation in heart failure with preserved or reduced ejection fraction: implications of distinct pathophysiologies on response to therapy. J Am Coll Cardiol 2012;59:442–451. 22281246 10.1016/j.jacc.2011.09.062

[56] Scrutinio D, Guida P, La Rovere MT, Bussotti M, Corrà U, Forni G, Raimondo R, Scalvini S, Passantino A. Functional outcome after cardiac rehabilitation and its association with survival in heart failure across the spectrum of ejection fraction. Eur J Intern Med 2023;110:86–92. 36759307 10.1016/j.ejim.2023.02.002

[57] Shah SJ, Fine N, Garcia-Pavia P, Klein AL, Fernandes F, Weissman NJ, Maurer MS, Boman K, Gundapaneni B, Sultan MB, Elliott P. Effect of tafamidis on cardiac function in patients with transthyretin amyloid cardiomyopathy: a post hoc analysis of the ATTR-ACT randomized clinical trial. JAMA Cardiol 2024;9:25–34. 37966817 10.1001/jamacardio.2023.4147 PMC10652219

[58] Steding-Ehrenborg K, Hedström E, Carlsson M, Maksuti E, Broomé M, Ugander M, Magnusson M, Smith JG, Arheden H. Hydraulic force is a novel mechanism of diastolic function that may contribute to decreased diastolic filling in HFpEF and facilitate filling in HFrEF. J Appl Physiol (1985) 2021;130:993–1000. 33539261 10.1152/japplphysiol.00890.2020

[59] Steding-Ehrenborg K, Nelsson A, Hedström E, Engblom H, Ingvarsson A, Nilsson J, Braun O, Arheden H. Diastolic filling in patients after heart transplantation is impaired due to an altered geometrical relationship between the left atrium and ventricle. J Am Heart Assoc 2024;13:e033672. 38780152 10.1161/JAHA.123.033672 PMC11255639

[60] Sugimoto T, Barletta M, Bandera F, Generati G, Alfonzetti E, Rovida M, Gnecchi Ruscone T, Rossi A, Cicoira M, Guazzi M. Central role of left atrial dynamics in limiting exercise cardiac output increase and oxygen uptake in heart failure: insights by cardiopulmonary imaging. Eur J Heart Fail 2020;22:1186–1198. 32352628 10.1002/ejhf.1829

[61] Vale-Lira A, Turri-Silva N, Verboven K, Durigan JLQ, de Lima ACGB, Bottaro M, Chiappa GR, Hansen D, Cipriano G. Muscle-skeletal abnormalities and muscle oxygenation during isokinetic strength exercise in heart failure with preserved ejection fraction phenotype: a cross-sectional study. Int J Environ Res Public Health 2022;19:709. 35055531 10.3390/ijerph19020709 PMC8775635

[62] Van Iterson EH, Johnson BD, Borlaug BA, Olson TP. Physiological dead space and arterial carbon dioxide contributions to exercise ventilatory inefficiency in patients with reduced or preserved ejection fraction heart failure. Eur J Heart Fail 2017;19:1675–1685. 28990307 10.1002/ejhf.913 PMC5741513

[63] Vuckovic KM, DeVon HA, Piano MR. Measurement of dyspnea in ambulatory African Americans with heart failure and a preserved or reduced ejection fraction. J Cardiovasc Nurs 2016;31:13–21. 25419941 10.1097/JCN.0000000000000205

[64] Wang T, Yu F-C, Wei Q, Chen L, Xu X, Ding N, Tong J-Y. Prevalence and clinical characteristics of sleep-disordered breathing in patients with heart failure of different left ventricular ejection fractions. Sleep Breath 2023;27:245–253. 35394577 10.1007/s11325-022-02611-4

[65] Warraich HJ, Kitzman DW, Whellan DJ, Duncan PW, Mentz RJ, Pastva AM, Nelson MB, Upadhya B, Reeves GR. Physical function, frailty, cognition, depression, and quality of life in hospitalized adults≥ 60 years with acute decompensated heart failure with preserved versus reduced ejection fraction: insights from the REHAB-HF trial. Circ Heart Fail 2018;11:e005254. 30571197 10.1161/CIRCHEARTFAILURE.118.005254 PMC6380360

[66] Wernhart S, Papathanasiou M, Rassaf T, Luedike P. Heart failure classification based on resting ejection fraction does not display a unique exercise response pattern. Int J Cardiol 2023;376:157–164. 36716970 10.1016/j.ijcard.2023.01.072

[67] Wisniacki N, Taylor W, Lye M, Wilding J. Insulin resistance and inflammatory activation in older patients with systolic and diastolic heart failure. Heart 2005;91:32–37. 15604330 10.1136/hrt.2003.029652 PMC1768659

[68] Zile MR, Mehra MR, Ducharme A, Sears SF, Desai AS, Maisel A, Paul S, Smart F, Grafton G, Kumar S, Nossuli TO, Johnson N, Henderson J, Adamson PB, Costanzo MR, Lindenfeld J. Hemodynamically-guided management of heart failure across the ejection fraction spectrum: the GUIDE-HF trial. Heart Failure 2022;10:931–944. 36456066 10.1016/j.jchf.2022.08.012

[69] Chelsea M, Costas T, Michelle S, John B, Theocharis I. Exercise-based cardiac rehabilitation: is a little encouragement enough? J Cardiopulm Rehabil Prev 2022;42:E97–E98. 36203219 10.1097/HCR.0000000000000736

[70] Meys R, Janssen S, Franssen FME, Vaes AW, Stoffels AAF, van Hees HWH, van den Borst B, Klijn PH, Burtin C, van't Hul AJ, Spruit MA. Test-retest reliability, construct validity and determinants of 6-minute walk test performance in adult patients with asthma. Pulmonology 2023;29:486–494. 36470816 10.1016/j.pulmoe.2022.10.011

[71] Uszko-Lencer NH, Mesquita R, Janssen E, Werter C, Brunner-La Rocca H-P, Pitta F, Wouters EFM, Spruit MA. Reliability, construct validity and determinants of 6-minute walk test performance in patients with chronic heart failure. Int J Cardiol 2017;240:285–290. 28377186 10.1016/j.ijcard.2017.02.109

[72] Swank AM, Horton J, Fleg JL, Fonarow GC, Keteyian S, Goldberg L, Wolfel G, Handberg EM, Bensimhon D, Illiou M-C, Vest M, Ewald G, Blackburn G, Leifer E, Cooper L, Kraus WE. Modest increase in peak VO2 is related to better clinical outcomes in chronic heart failure patients: results from heart failure and a controlled trial to investigate outcomes of exercise training. Circ Heart Fail 2012;5:579–585. 22773109 10.1161/CIRCHEARTFAILURE.111.965186 PMC3732187

[73] Wessler BS, Kramer DG, Kelly JL, Trikalinos TA, Kent DM, Konstam MA, Udelson JE. Drug and device effects on peak oxygen consumption, 6-minute walk distance, and natriuretic peptides as predictors of therapeutic effects on mortality in patients with heart failure and reduced ejection fraction. Circ Heart Fail 2011;4:578–588. 21705485 10.1161/CIRCHEARTFAILURE.111.961573

[74] Psotka MA, Abraham WT, Fiuzat M, Filippatos G, Lindenfeld J, Ahmad T, Felker GM, Jacob R, Kitzman DW, Leifer ES, Lewis EF, Mentz RJ, Nkulikiyinka R, Ni W, Schaber DE, Sharma A, Solomon SD, Stockbridge N, Teerlink JR, Unger EF, Whellan DJ, Wittes J, Anker SD, O’Connor CM. Functional and symptomatic clinical trial endpoints: the HFC-ARC scientific expert panel. JACC Heart Fail 2022;10:889–901. 36456063 10.1016/j.jchf.2022.09.012

[75] Nadruz W, West E Jr, Sengeløv M, Santos M, Groarke JD, Forman DE, Claggett B, Skali H, Shah AM. Prognostic value of cardiopulmonary exercise testing in heart failure with reduced, midrange, and preserved ejection fraction. J Am Heart Assoc 2017;6:e006000. 29089342 10.1161/JAHA.117.006000 PMC5721737

[76] Taylor RS, Sagar VA, Davies EJ, Briscoe S, Coats AJ, Dalal H, Lough F, Rees K, Singh S. Exercise-based rehabilitation for heart failure. Cochrane Database Syst Rev 2014;4:CD003331. 10.1002/14651858.CD003331.pub4 PMC6485909 24771460

[77] Long L, Mordi IR, Bridges C, Sagar VA, Davies EJ, Coats AJ, Dalal H, Rees K, Singh SJ, Taylor RS. Exercise-based cardiac rehabilitation for adults with heart failure. Cochrane Database Syst Rev 2019;1:CD003331. 30695817 10.1002/14651858.CD003331.pub5 PMC6492482

[78] Cipriano G Jr, da Luz Goulart C, Chiappa GR, da Silva ML, Silva NT, do Vale Lira AO, Negrão EM, DÁvila LBO, Ramalho SHR, de Souza FSJ, Cipriano GFB, Hirai D, Hansen D, Cahalin LP. Differential impacts of body composition on oxygen kinetics and exercise tolerance of HFrEF and HFpEF patients. Sci Rep 2024;14:22505. 39341902 10.1038/s41598-024-72965-0 PMC11439022

[79] D’Ávila LBO, de Lima ACGB, Milani M, Milani JGPO, Cipriano GFB, Le Bihan DCS, Castro I, Cipriano G Jr. Left ventricular global longitudinal strain and cardiorespiratory fitness in patients with heart failure: systematic review and meta-analysis. Hellenic J Cardiol 2024;79:58–69. 37778639 10.1016/j.hjc.2023.09.010

[80] Chaanine AH, Joyce LD, Stulak JM, Maltais S, Joyce DL, Dearani JA, Klaus K, Nair KS, Hajjar RJ, Redfield MM. Mitochondrial morphology, dynamics, and function in human pressure overload or ischemic heart disease with preserved or reduced ejection fraction. Circ Heart Fail 2019;12:e005131. 30744415 10.1161/CIRCHEARTFAILURE.118.005131

