# Supplementary material for: Exercise capacity in heart failure: a systematic review and meta-analysis of HFrEF and HFpEF disparities in VO2peak and 6-minute walking distance
Source: Eur Heart J Open. 2025 May 14;5(3):oeaf055. doi: 10.1093/ehjopen/oeaf055 (PMC12202100; doi:10.1093/ehjopen/oeaf055)
Supplement: oeaf055_Supplementary_Data [file oeaf055_supplementary_data.zip › Supplementary Materials.docx]

**Vo2peak – Proportion of females to total sample**


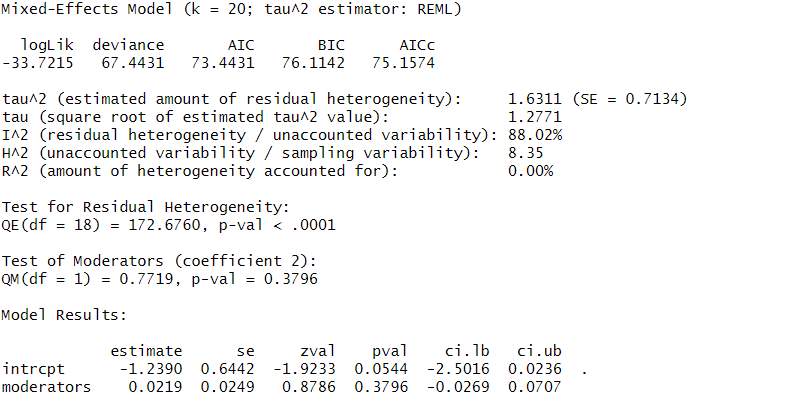


**Vo2peak – BMI**


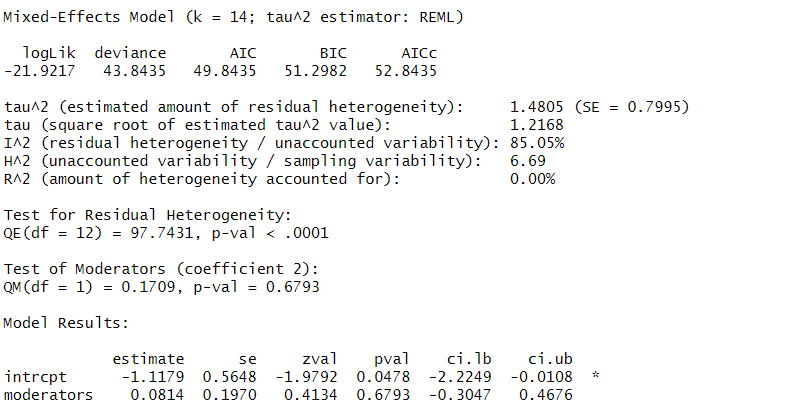


**Vo2peak – Age**

**
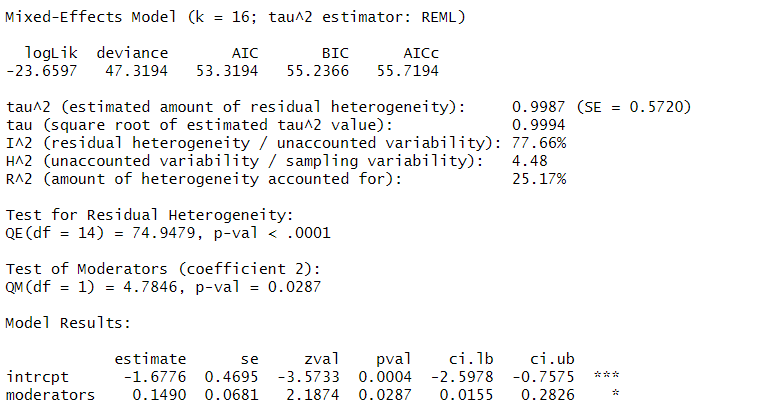
**

**Stroke volume – Proportion of females to total sample**

**
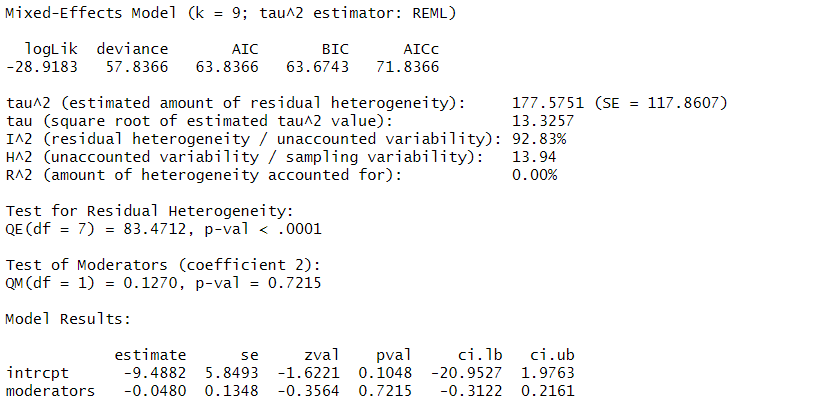
**

**Stroke volume – BMI**

**
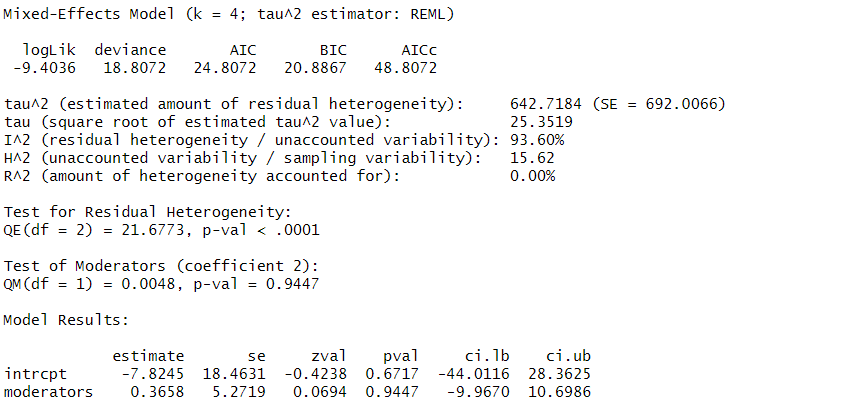
**

**Stroke Volume – Age**

**
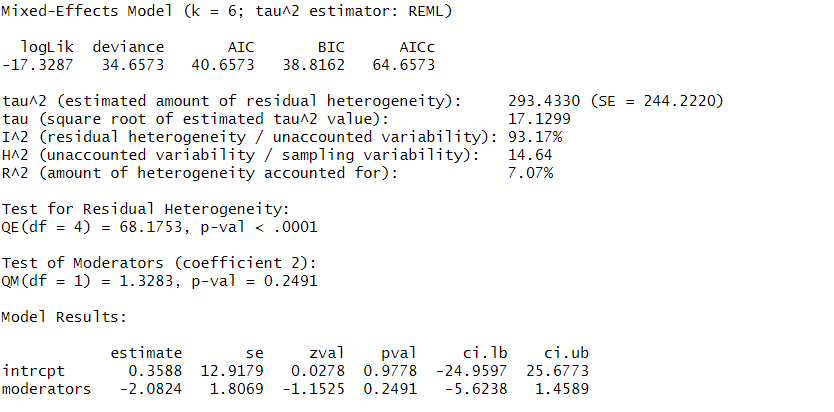
**

**Cardiac Output – Proportion of females to total sample**

**
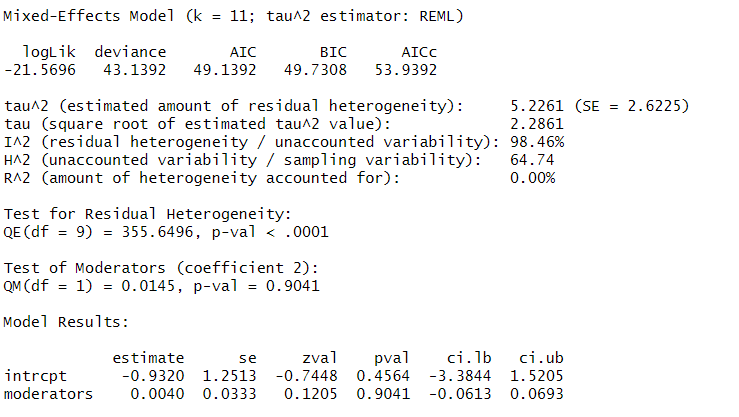
**

**Cardiac Output – BMI**

**
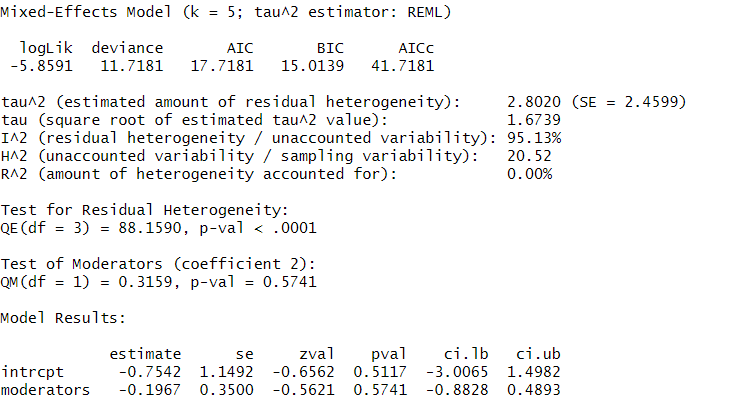
**

**Cardiac Output – Age**

**
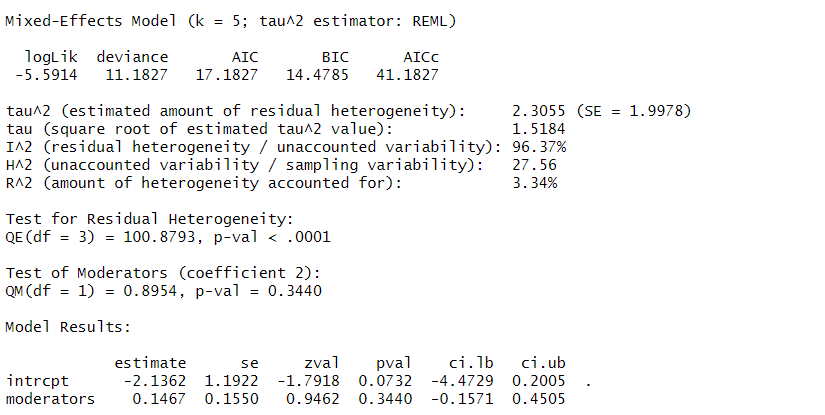
**

**6MWD - Cardiac Output – Proportion of females to total sample**

**
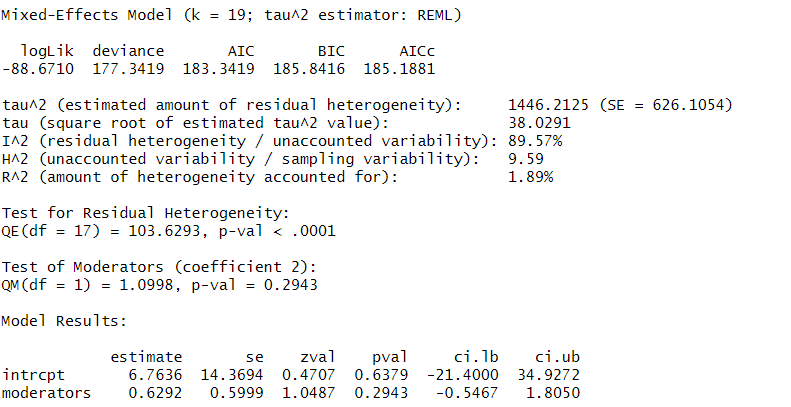
**

**6MWD – BMI**

**
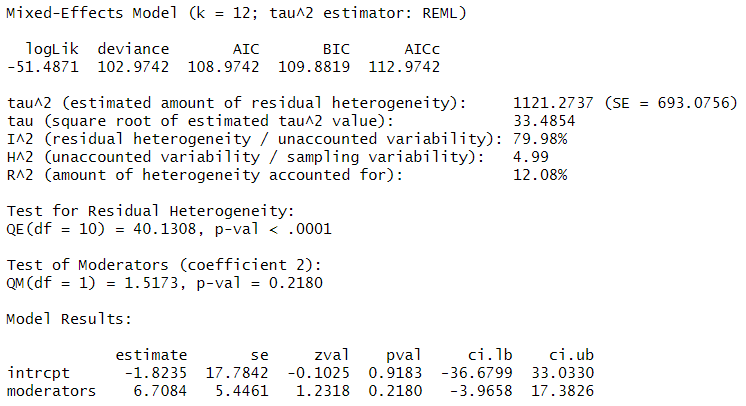
**

**6MWD – Age**

**
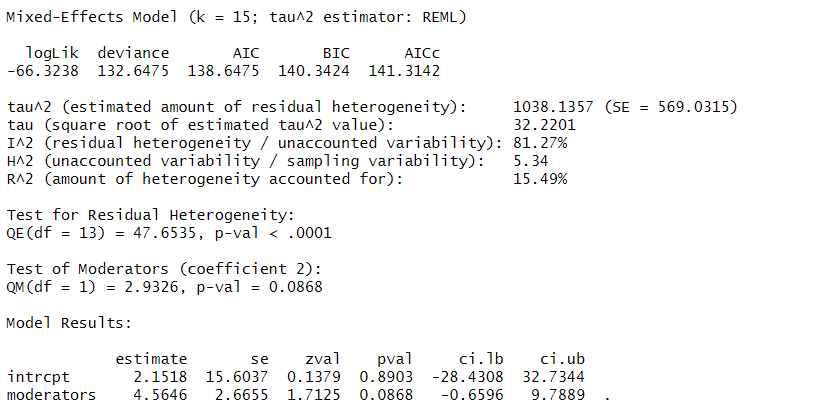
**

**Vo2peak – Publication bias**

**
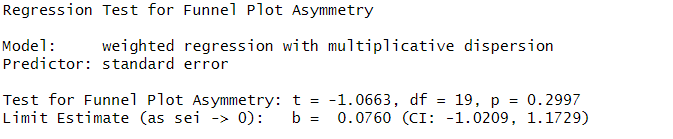
**

**
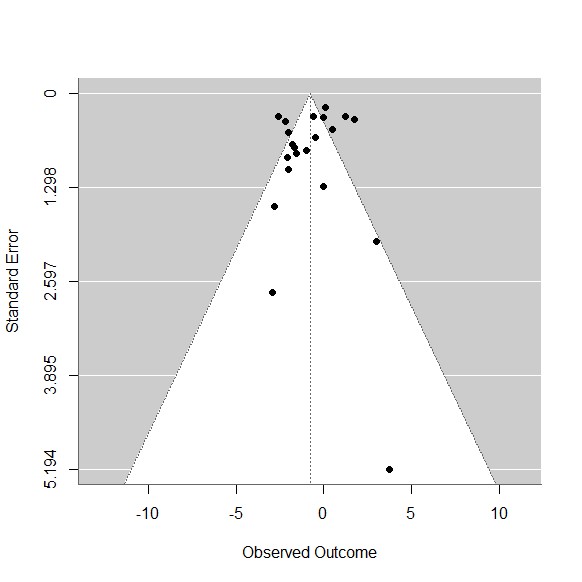
**

**Stroke Volume – Publication bias**

**
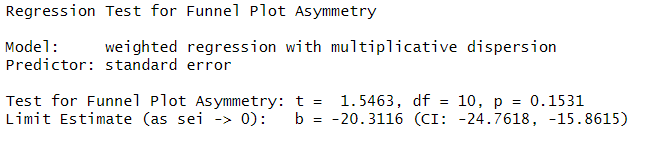
**

**
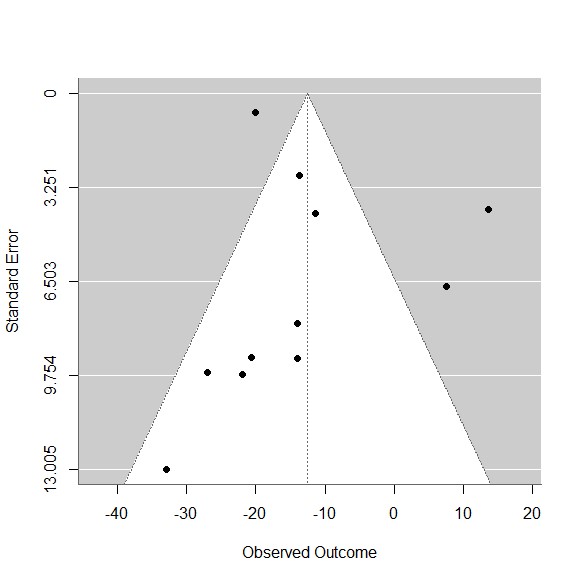
**

**Cardiac Output – Publication bias**

**
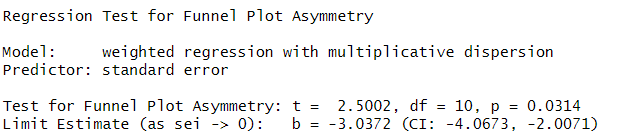
**

**
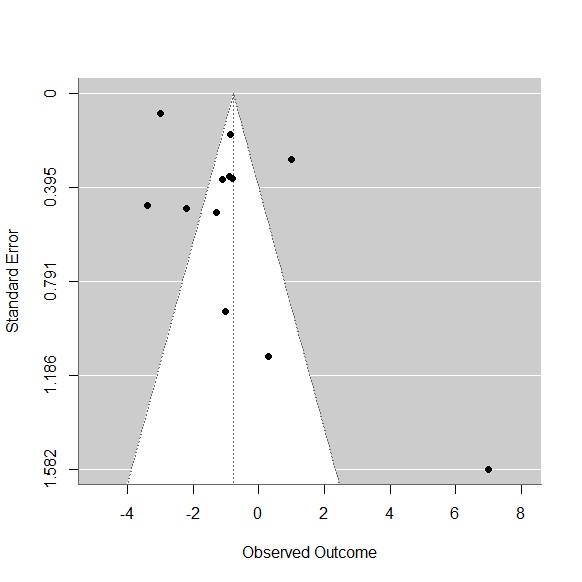
**

**6MWD – Publication bias**

**
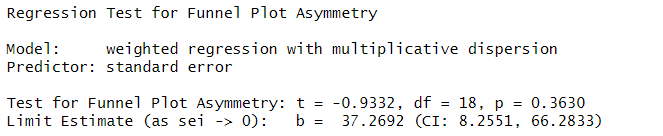
**

**
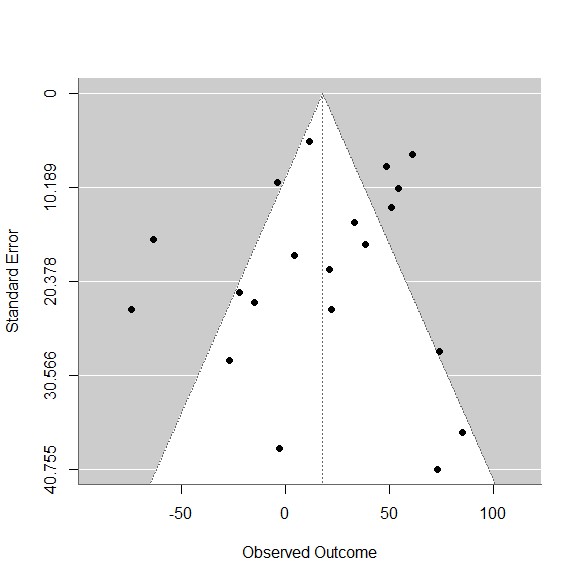
**
