# Supplementary material for: Exercise capacity in heart failure: a systematic review and meta-analysis of HFrEF and HFpEF disparities in VO2peak and 6-minute walking distance
Source: Eur Heart J Open. 2025 May 14;5(3):oeaf055. doi: 10.1093/ehjopen/oeaf055 (PMC12202100; doi:10.1093/ehjopen/oeaf055)
Supplement: oeaf055_Supplementary_Data [file oeaf055_supplementary_data.zip › Table S1.docx]

**Table S1.**Search terms employed in the screening based on title, abstract, and keywords in the literature search.

| **Database** | **Search terms** |
| --- | --- |
|  |  |
| PubMed | (“HFpEF” AND “HFrEF”) OR (“heart failure with preserved ejection fraction” AND “heart failure with reduced ejection fraction”)  AND (“cardiac output” OR “stroke volume” OR 6-minute walk* OR “Vo2peak” OR “Vo2max” OR “maximal oxygen capacity”  OR “maximal oxygen consumption”) |
| Cochrane Library | (“HFpEF” AND “HFrEF”) OR (“heart failure with preserved ejection fraction” AND “heart failure with reduced ejection fraction”)  AND (“cardiac output” OR “stroke volume” OR 6-minute walk* OR “Vo2peak” OR “Vo2max” OR “maximal oxygen capacity”  OR “maximal oxygen consumption”) |
| Web of Science | (“HFpEF” AND “HFrEF”) OR (“heart failure with preserved ejection fraction” AND “heart failure with reduced ejection fraction”)  AND (“cardiac output” OR “stroke volume” OR 6-minute walk* OR “Vo2peak” OR “Vo2max” OR “maximal oxygen capacity”  OR “maximal oxygen consumption”) |
| Scopus | (“HFpEF” AND “HFrEF”) OR (“heart failure with preserved ejection fraction” AND “heart failure with reduced ejection fraction”)  AND (“cardiac output” OR “stroke volume” OR 6-minute walk* OR “Vo2peak” OR “Vo2max” OR “maximal oxygen capacity”  OR “maximal oxygen consumption”) |
