# Supplementary material for: Exercise capacity in heart failure: a systematic review and meta-analysis of HFrEF and HFpEF disparities in VO2peak and 6-minute walking distance
Source: Eur Heart J Open. 2025 May 14;5(3):oeaf055. doi: 10.1093/ehjopen/oeaf055 (PMC12202100; doi:10.1093/ehjopen/oeaf055)
Supplement: oeaf055_Supplementary_Data [file oeaf055_supplementary_data.zip › Table S2.docx]

**Table S2.** Methods of Peak VO_2_ and left ventricular ejection fraction assessment in the included studies.

| **Study** | **Peak VO_2_ assessment method** | **LVEF assessment method** |
| --- | --- | --- |
| Abe 2013 | n/a | Echocardiography |
| Adams 2021 | An incremental protocol until exhaustion on a bicycle ergometer. The protocol comprised a 10 Watt increase in workload every minute, starting at 20 Watt. In control subjects, workload was increased progressively every 3 min in steps of 25 Watt. The mean of the three highest 10 s consecutive measurements was identified as peak VO_2_. | Echocardiography |
| Arvidsson 2022 | n/a | Both echocardiography and cMRI were done, but authors did not report which method was used to obtain LVEF. |
| Bekfani 2020 | n/a | Not reported |
| Blum 2020 | n/a | cMRI |
| Charman 2022 | n/a | Echocardiography |
| Chung 2008 | A modified Bruce treadmill protocol. | Echocardiography |
| Conti 2020 | n/a | Echocardiography |
| Daubert 2018 (OMT alone) | n/a | Echocardiography |
| de Denus 2012 | n/a | Echocardiography or ventriculography |
| Dhakal 2015 | n/a | Ventriculography |
| Edlund 2022 | n/a | cMRI |
| Fudim 2020 | A custom treadmill protocol was designed with a linear increase in walking speed coupled with a curvilinear increase in treadmill grade to yield a linear increase in work rate of 10W/min. Peak VO_2_ is determined by the highest 30-second median value of breath-by-breath VO_2_ measurements during the final minute of incremental exercise. | Echocardiography |
| Fujiwara 2021 | Cycle ergometry with a ramp protocol. Peak VO_2_ was defined as the highest VO_2_ value. | Echocardiography |
| Gong 2022 | Cycle ergometry with a ramp protocol. Peak VO_2_ was defined as the highest 10 s averaged VO_2_ around the time of maximal effort. | LVEF derived either by transthoracic echocardiography (n = 1309) or cardiac magnetic resonance imaging (n = 38) |
| Guazzi 2014 | An upright graded bicycle exercise using a personalized ramp protocol. Peak VO_2_ was defined as the highest 30-second averaged value obtained during exercise. | Echocardiography |
| Hou 2023 | n/a | cMRI |
| Hsu 2024 (GDMT group) | An incremental protocol on bicycle ergometry, consisting of 2 min of unloaded pedaling followed by a continuous increase in work-rate of 10 W per min until exhaustion. | Echocardiography |
| Hsu 2024 (HIIT group) | An incremental protocol on bicycle ergometry, consisting of 2 min of unloaded pedaling followed by a continuous increase in work-rate of 10 W per min until exhaustion. | Echocardiography |
| Hundley 2007 | Upright stationary bicycle exercise began at an initial workload of 12.5 W, was advanced to 25 W 2 min into exercise, and was then increased by 25-W increments every 3 min thereafter to the point of exhaustion. Peak oxygen consumption was considered to be the highest oxygen consumption achieved during exercise. | Not reported |
| Ingle 2015 | n/a | Echocardiography |
| Kanagala 2020 | n/a | Echocardiography |
| Li 2024 | n/a | Echocardiography |
| Luo 2020 | The revised Ramp10 program with bicycle ergometry was adopted, rest on the cycle for 3 minutes, cycle for 3 minutes under no load, and then start from 0 J/second, increase 5 J every 30 seconds until the patient reaches a peak of exercise or the end of exercise. | Echocardiography |
| Maldonado-Martin 2005 | n/a | Echocardiography |
| Moriwaki 2021 | Maximal symptom-limited cardiopulmonary exercise was performed using a cycle ergometer with a ramp protocol with increments of 1 W per 6 s until exhaustion. | Echocardiography |
| Namasivayam 2022 | Upright maximum incremental ramp exercise testing was performed using a cycle ergometer. After a resting period ≥3 min, there was a period of unloaded exercise for 3 min and then a continuous ramp (5-30 W/min, based on estimated exercise capacity) designed to achieve 8-12 min of total exercise at a cadence of 60 revolutions/min. | Echocardiography or ventriculography – details not reported |
| Obokata 2017 | n/a | Echocardiography |
| Paolisso 2024 | n/a | Echocardiography |
| Pugliese 2019 | A symptom-limited graded ramp bicycle exercise test was performed in the semi-supine position on a tilting, dedicated, stress echocardiography cycle ergometer. Authors estimated the expected peak oxygen consumption based on patient age, height, weight, and clinical history. Then the work rate increment was calculated necessary to reach the patient’s estimated peak VO_2_ in 8–12 min. The protocol included 2 min of unloaded pedalling and 4 min of recovery after peak effort. Peak VO_2_ was the highest averaged 30-s VO_2_ during exercise. | Echocardiography |
| Rickenbacher 2017 | n/a | Echocardiography |
| Sato 2003 | A symptom-limited graded exercise with a 4-min stage using a supine bicycle ergometer. The workload was increased by 15–25 W according to individual exercise capacity. | Ventriculography |
| Sato 2017 | An incremental symptom-limited exercise testing using an upright cycle ergometer with a ramp protocol. Peak VO_2_ was measured as an average of the last 30 s of exercise. | Echocardiography |
| Schwartzenberg 2012 | n/a | Echocardiography |
| Scrutinio 2023 | n/a | Echocardiography |
| Shah 2024 | n/a | Echocardiography |
| Steding-Ehrenborg 2021 | n/a | cMRI |
| Steding-Ehrenborg 2024 | n/a | Echocardiography and cMRI |
| Sugimoto 2020 | A symptom-limited CPET was performed on a semisupine position by tilt test ergometry in all subjects. Incremental ramp protocols were designed to obtain standard of exercise. The ramp steep was limited to a maximum of 15W/min to facilitate simultaneous echocardiographic assessment. Peak VO_2_ and peak respiratory exchange ratio were expressed as the averaged sample obtained during the final 20s. | Echocardiography |
| Vale-Lira 2022 | Functional exercise capacity was assessed utilizing a maximal incremental cardiopulmonary exercise test of an electromagnetic bicycle. 1-minute work stage protocol (starting workload of 20W and incremental workload of 10 to 15 W). | Echocardiography |
| Van Iterson 2017 | Patients exercised in a semi-recumbent position on a cycle ergometer at an initial workload of 20-watts while pedaling at 60–65 rpm, thereafter increasing by 10-W every 3 min until volitional fatigue. | Echocardiography |
| Vuckovic 2016 | n/a | Echocardiography |
| Wang 2023 | n/a | Echocardiography |
| Warraich 2018 | n/a | Echocardiography, MRI, cardiac catheterization, or nuclear medicine scan – details not reported |
| Wernhart 2023 | A ramp protocol with an exercise duration of 8–12 min. Every exercise test was performed until maximal exertion defined as a respiratory exchange ratio >1.05. | Echocardiography |
| Wisniacki 2005 | n/a | Echocardiography |
| Zile 2022 | n/a | Not reported |
