# Supplementary material for: Exercise capacity in heart failure: a systematic review and meta-analysis of HFrEF and HFpEF disparities in VO2peak and 6-minute walking distance
Source: Eur Heart J Open. 2025 May 14;5(3):oeaf055. doi: 10.1093/ehjopen/oeaf055 (PMC12202100; doi:10.1093/ehjopen/oeaf055)
Supplement: oeaf055_Supplementary_Data [file oeaf055_supplementary_data.zip › Table S3.docx]

**Table S3.** Publication bias through Egger’s test for each outcome.

|  | ***p*** | ***t*** | ***b*** | **95%CI** |
| --- | --- | --- | --- | --- |
| **VO_2_max** | 0.30 | -1.0663 | 0.076 | -1.02 – 1.17 |
| **Stroke Volume** | 0.15 | 1.5463 | -20.312 | -24.76 – -15.86 |
| **Cardiac Output** | 0.03* | 2.5002 | -3.037 | -4.07 – -2.01 |
| **6-minute walking distance** | 0.36 | -0.9332 | 37.269 | 8.26 – 66.28 |

*Indicates significance, p < 0.05.
