# Supplementary material for: Exercise capacity in heart failure: a systematic review and meta-analysis of HFrEF and HFpEF disparities in VO2peak and 6-minute walking distance
Source: Eur Heart J Open. 2025 May 14;5(3):oeaf055. doi: 10.1093/ehjopen/oeaf055 (PMC12202100; doi:10.1093/ehjopen/oeaf055)
Supplement: oeaf055_Supplementary_Data [file oeaf055_supplementary_data.zip › Table S4.docx]

**Table S4.** Meta-regression analyses evaluating sex, BMI, and age as potential moderators.

|  | ***z*** | ***se*** | ***p*** | **95%CI** |
| --- | --- | --- | --- | --- |
| **VO_2_max** | | | | |
| Proportion of females | 0.88 | 0.02 | 0.38 | -0.03 – 0.07 |
| BMI | 0.41 | 0.20 | 0.68 | -0.30 – 0.47 |
| Age | 2.19 | 0.07 | 0.03* | 0.02 – 0.28 |
| **Stroke Volume** | | | | |
| Proportion of females | -0.36 | 0.13 | 0.72 | -0.31 – 0.22 |
| BMI | 0.07 | 5.27 | 0.94 | -9.97 – 10.70 |
| Age | -1.15 | 1.81 | 0.25 | -5.62 – 1.46 |
| **Cardiac Output** | | | | |
| Proportion of females | 0.12 | 0.03 | 0.90 | -0.06 – 0.07 |
| BMI | -0.56 | -0.35 | 0.57 | -0.88 – 0.49 |
| Age | 0.95 | 0.16 | 0.34 | -0.16 – 0.45 |
| **6-minute walking distance** | | | | |
| Proportion of females | 1.05 | 0.60 | 0.29 | -0.55 – 1.81 |
| BMI | 1.23 | 5.45 | 0.22 | -3.97 – 17.38 |
| Age | 1.71 | 2.67 | 0.09 | -0.66 – 9.79 |

*Indicates significance, p < 0.05.
BMI, body mass index.
