# Supplementary material for: Exercise capacity in heart failure: a systematic review and meta-analysis of HFrEF and HFpEF disparities in VO2peak and 6-minute walking distance
Source: Eur Heart J Open. 2025 May 14;5(3):oeaf055. doi: 10.1093/ehjopen/oeaf055 (PMC12202100; doi:10.1093/ehjopen/oeaf055)
Supplement: oeaf055_Supplementary_Data [file oeaf055_supplementary_data.zip › Table S6.docx]

| **Table S6.** NOS for the risk of bias and quality assessment of included cohort studies. | | | | | | | | | |  |
| --- | --- | --- | --- | --- | --- | --- | --- | --- | --- | --- |
| Author, Year | Selection | | | | Comparability | Outcome | | | Total score (maximum 9 possible) | Overall |
|  | Representativeness of the exposed cohort | Selection of the non-exposed cohort | Ascertainment of exposure | Demonstration that outcome of interest was not present at start of study | Control for important or additional factors | Assessment of outcome | Was follow up long enough for outcomes to occur | Adequacy of follow up of cohorts |  |  |
| Abe 2013 |  | **⋆** | **⋆** | **⋆** | **⋆** | **⋆** | **⋆** | **⋆** | 7 | Moderate |
| Fudim 2020 | **⋆** |  | **⋆** | **⋆** | **⋆** | **⋆** | **⋆** |  | 6 | Moderate |
| Gong 2022 | **⋆** | **⋆** | **⋆** | **⋆** | **⋆** | **⋆** | **⋆** | **⋆** | 8 | Low |
| Hsu 2024 | **⋆** |  | **⋆** | **⋆** | **⋆⋆** | **⋆** | **⋆** | **⋆** | 8 | Low |
| Kangala 2020 | **⋆** |  | **⋆** | **⋆** | **⋆⋆** | **⋆** | **⋆** | **⋆** | 8 | Low |
| Moriwaki 2021 | **⋆** |  | **⋆** | **⋆** |  | **⋆** | **⋆** | **⋆** | 6 | Moderate |
| Namasivayam 2022 | **⋆** | **⋆** | **⋆** | **⋆** | **⋆⋆** | **⋆** | **⋆** | **⋆** | 9 | Low |
| Rickenbacher 2017 | **⋆** | **⋆** | **⋆** | **⋆** |  | **⋆** | **⋆** | **⋆** | 7 | Moderate |
| Sato 2017 | **⋆** | **⋆** | **⋆** | **⋆** | **⋆⋆** | **⋆** | **⋆** | **⋆** | 9 | Low |
| Scrutinio 2023 | **⋆** | **⋆** | **⋆** | **⋆** | **⋆⋆** | **⋆** | **⋆** | **⋆** | 9 | Low |
| Sugimoto 2020 | **⋆** | **⋆** | **⋆** | **⋆** | **⋆⋆** | **⋆** | **⋆** | **⋆** | 9 | Low |
