# Supplementary material for: Exercise capacity in heart failure: a systematic review and meta-analysis of HFrEF and HFpEF disparities in VO2peak and 6-minute walking distance
Source: Eur Heart J Open. 2025 May 14;5(3):oeaf055. doi: 10.1093/ehjopen/oeaf055 (PMC12202100; doi:10.1093/ehjopen/oeaf055)
Supplement: oeaf055_Supplementary_Data [file oeaf055_supplementary_data.zip › Table S7.docx]

| **Table S7.** NOS for the risk of bias and quality assessment of included cross-sectional studies. | | | | | | | |  |
| --- | --- | --- | --- | --- | --- | --- | --- | --- |
| Author, Year | Selection | | | Comparability | Outcome | | Total score (maximum 7 possible) | Overall |
|  | Representativeness of the intervention cohort | Sample size | Ascertainment of exposure | Control for important or additional factors | Assessment of outcome | Statistical test |  |  |
| Adams 2021 |  |  | **⋆** |  | **⋆** | **⋆** | 3 | High |
| Arvidsson 2022 | **⋆** |  | **⋆** |  | **⋆** | **⋆** | 4 | Moderate |
| Blum 2020 |  |  | **⋆** |  | **⋆** | **⋆** | 4 | Moderate |
| Charman 2022 | **⋆** |  | **⋆** |  | **⋆** | **⋆** | 4 | Moderate |
| Chung 2008 |  |  | **⋆** |  | **⋆** | **⋆** | 3 | High |
| Conti 2020 | **⋆** |  | **⋆** | **⋆** | **⋆** | **⋆** | 5 | Moderate |
| de Denus 2012 | **⋆** |  | **⋆** | **⋆⋆** | **⋆** | **⋆** | 6 | Low |
| Dhakal 2015 |  |  | **⋆** | **⋆** | **⋆** | **⋆** | 4 | Moderate |
| Edlund 2022 | **⋆** |  | **⋆** |  | **⋆** | **⋆** | 4 | Moderate |
| Fujiwara 2021 | **⋆** | **⋆** | **⋆** | **⋆** | **⋆** | **⋆** | 6 | Low |
| Guazzi 2014 | **⋆** |  | **⋆** | **⋆⋆** | **⋆** | **⋆** | 6 | Low |
| Hundley 2007 | **⋆** |  | **⋆** | **⋆⋆** | **⋆** | **⋆** | 6 | Low |
| Ingle 2015 | **⋆** | **⋆** | **⋆** | **⋆** | **⋆** | **⋆** | 6 | Low |
| Li 2024 | **⋆** |  | **⋆** |  | **⋆** | **⋆** | 4 | Moderate |
| Maldonado-martin 2005 | **⋆** |  | **⋆** |  | **⋆** | **⋆** | 4 | Moderate |
| Obokata 2017 | **⋆** |  | **⋆** |  | **⋆** | **⋆** | 4 | Moderate |
| Pugliese 2019 | **⋆** |  | **⋆** | **⋆⋆** | **⋆** | **⋆** | 6 | Low |
| Sato 2003 |  |  | **⋆** |  | **⋆** | **⋆** | 3 | High |
| Schwartzenberg 2012 | **⋆** | **⋆** | **⋆** | **⋆⋆** | **⋆** | **⋆** | 7 | Low |
| Steding-Ehrenborg 2021 |  |  | **⋆** |  | **⋆** | **⋆** | 3 | High |
| Steding-Ehrenborg 2024 | **⋆** | **⋆** | **⋆** | **⋆⋆** | **⋆** | **⋆** | 7 | Low |
| Vale-Lira 2022 | **⋆** |  | **⋆** |  | **⋆** | **⋆** | 4 | Moderate |
| Van Iterson 2017 |  |  | **⋆** |  | **⋆** | **⋆** | 3 | High |
| Vuckovic 2016 | **⋆** |  | **⋆** | **⋆** | **⋆** | **⋆** | 5 | Moderate |
| Wang 2023 | **⋆** | **⋆** | **⋆** | **⋆** | **⋆** | **⋆** | 6 | Low |
| Warraich 2018 | **⋆** | **⋆** | **⋆** | **⋆** | **⋆** |  | 5 | Moderate |
| Wernhart 2023 | **⋆** | **⋆** | **⋆** | **⋆⋆** | **⋆** | **⋆** | 7 | Low |
| Wisniacki 2005 |  |  | **⋆** |  | **⋆** | **⋆** | 3 | High |
