# Supplementary figures and images for: Exercise capacity in heart failure: a systematic review and meta-analysis of HFrEF and HFpEF disparities in VO2peak and 6-minute walking distance
Source: Eur Heart J Open. 2025 May 14;5(3):oeaf055. doi: 10.1093/ehjopen/oeaf055 (PMC12202100; doi:10.1093/ehjopen/oeaf055)

**Table S5.** Risk of bias assessment of the included randomised controlled trials.


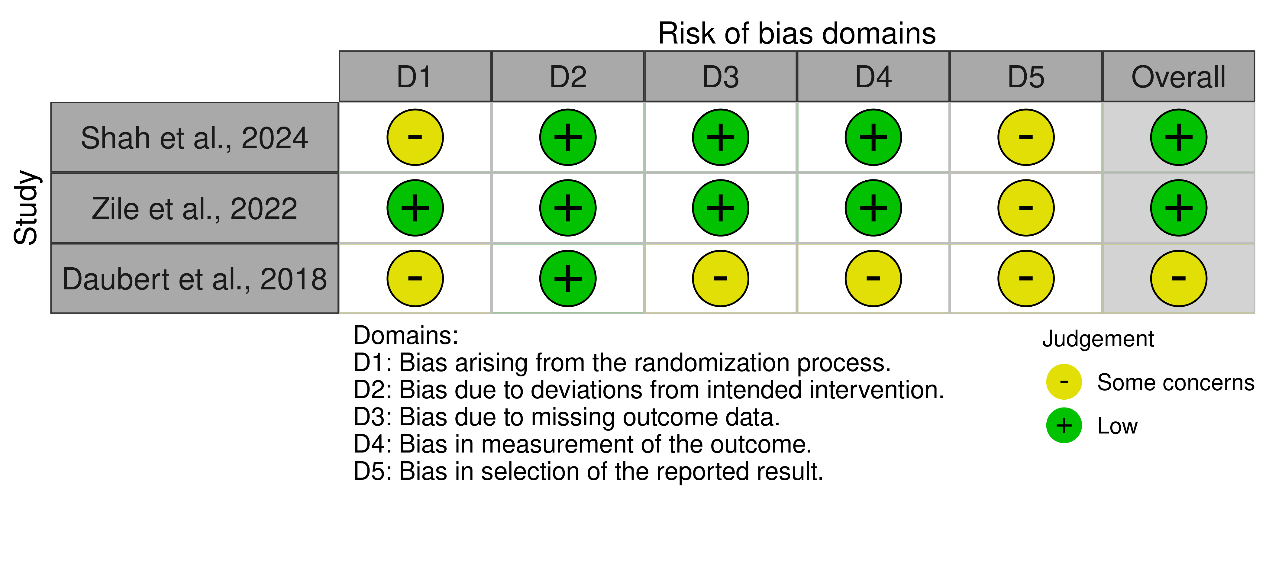

Supplement: oeaf055_Supplementary_Data [file oeaf055_supplementary_data.zip › Table S5.docx]

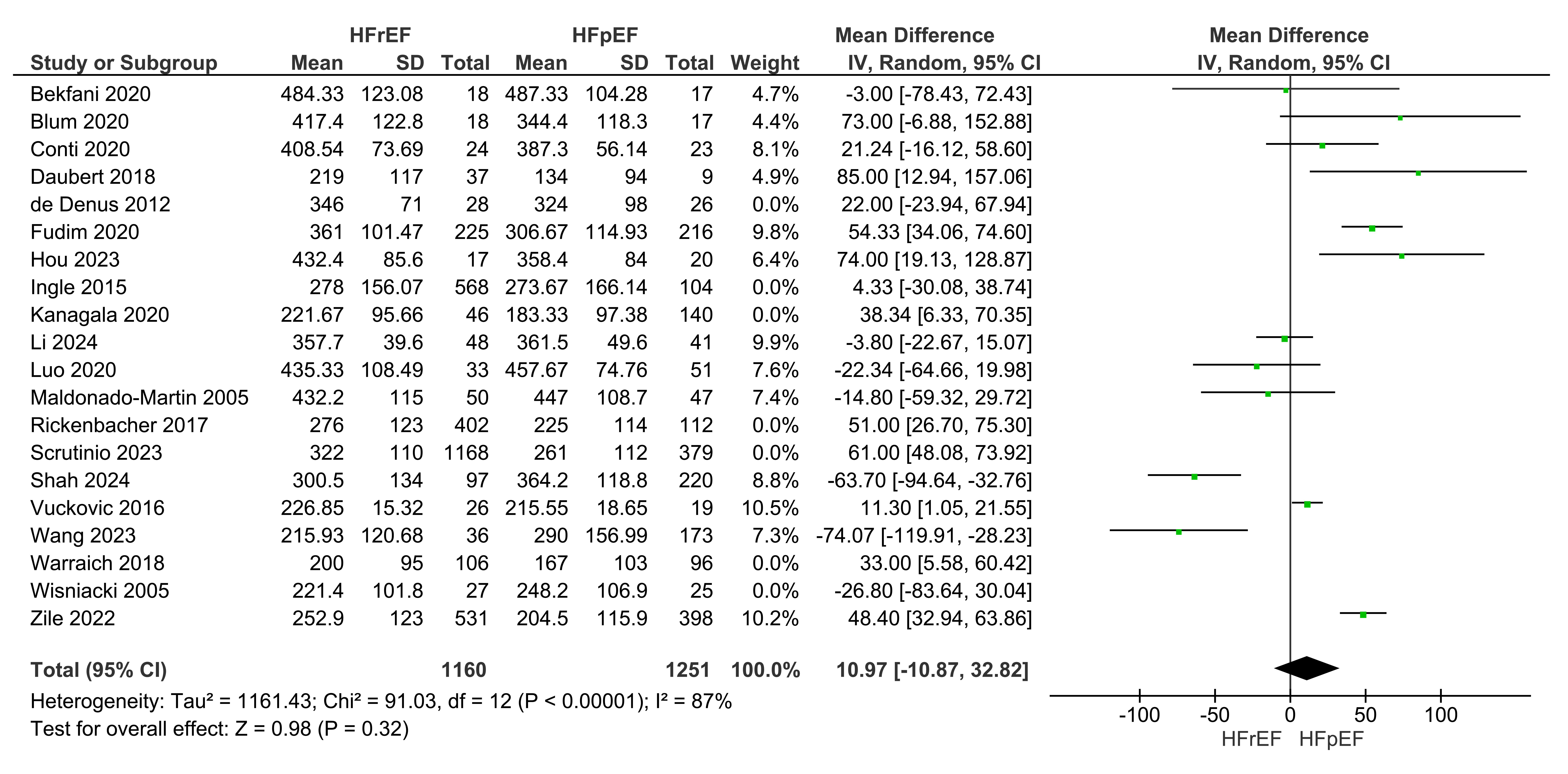

Supplement: oeaf055_Supplementary_Data [file oeaf055_supplementary_data.zip › Figure S1.tiff]

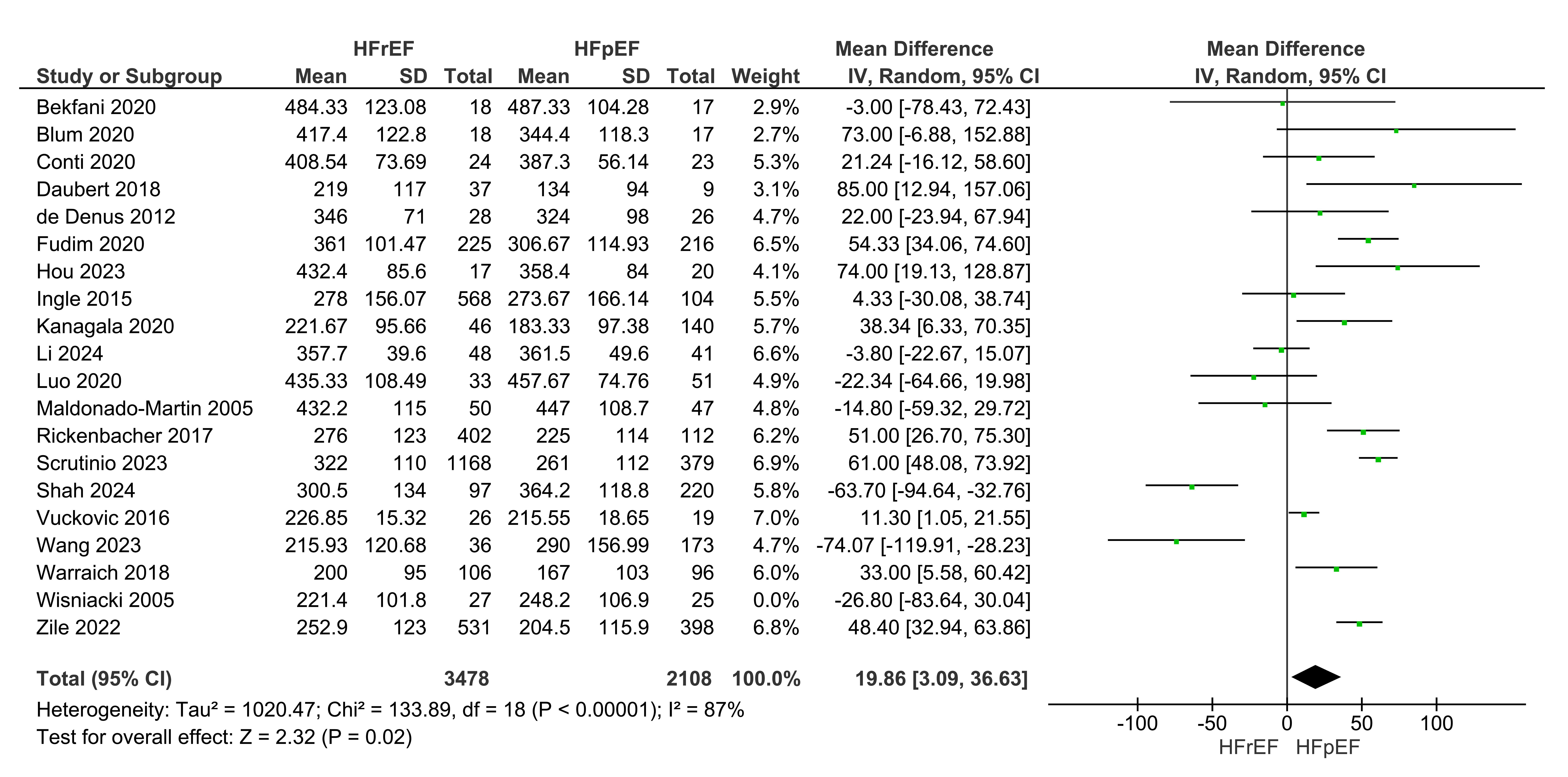

Supplement: oeaf055_Supplementary_Data [file oeaf055_supplementary_data.zip › Figure S2.tiff]

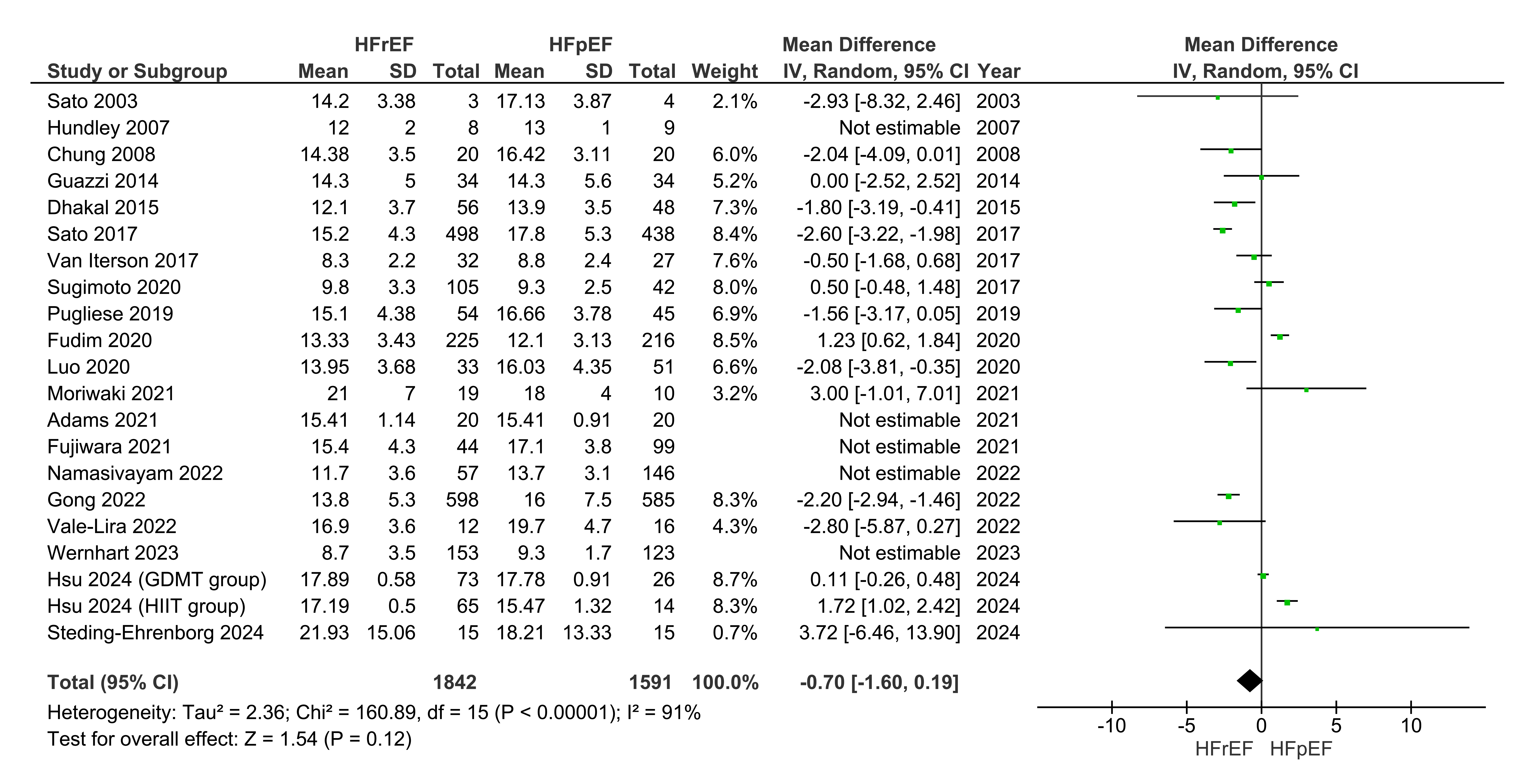

Supplement: oeaf055_Supplementary_Data [file oeaf055_supplementary_data.zip › Figure S3.tiff]

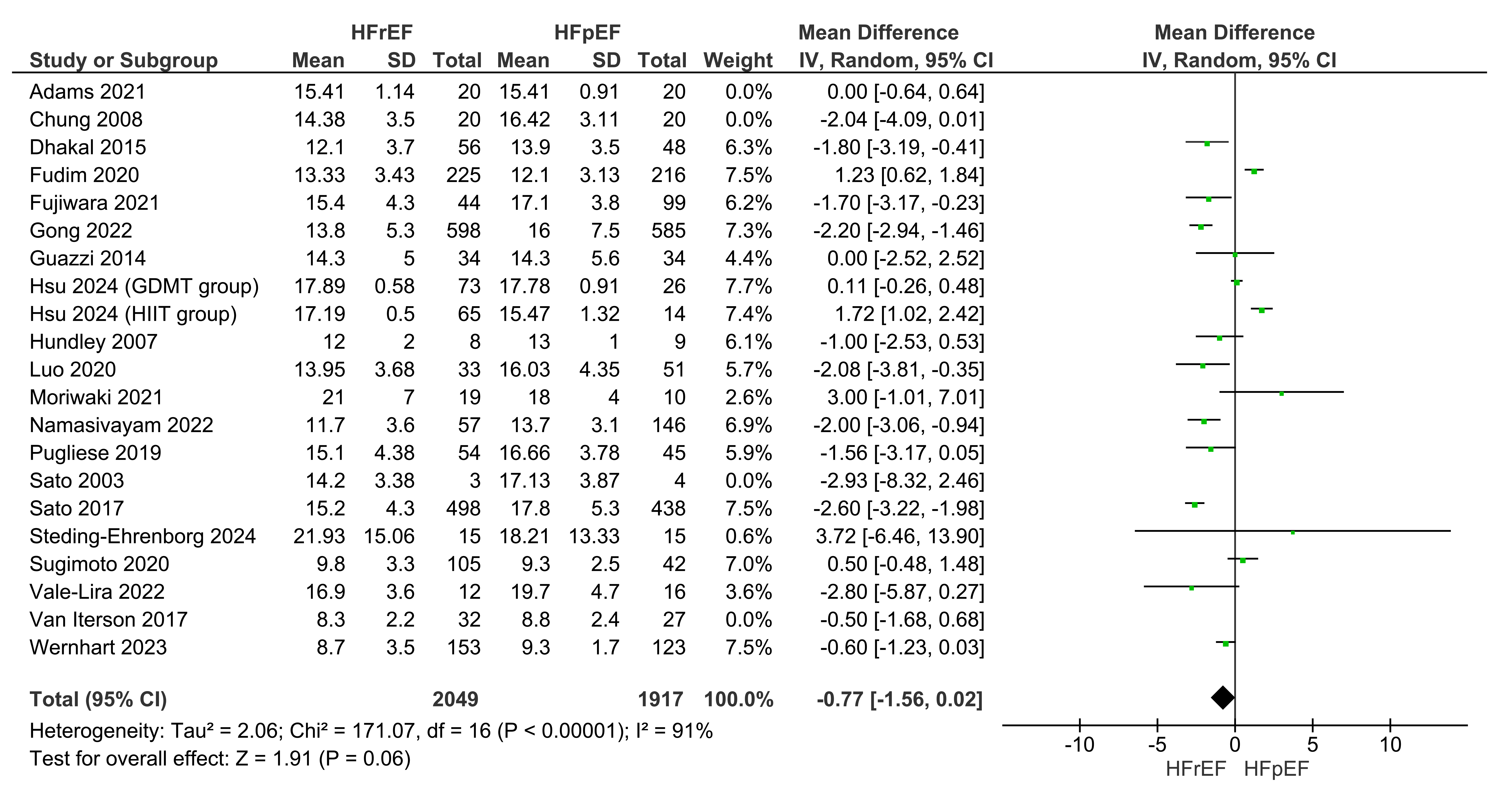

Supplement: oeaf055_Supplementary_Data [file oeaf055_supplementary_data.zip › Figure S4.tiff]

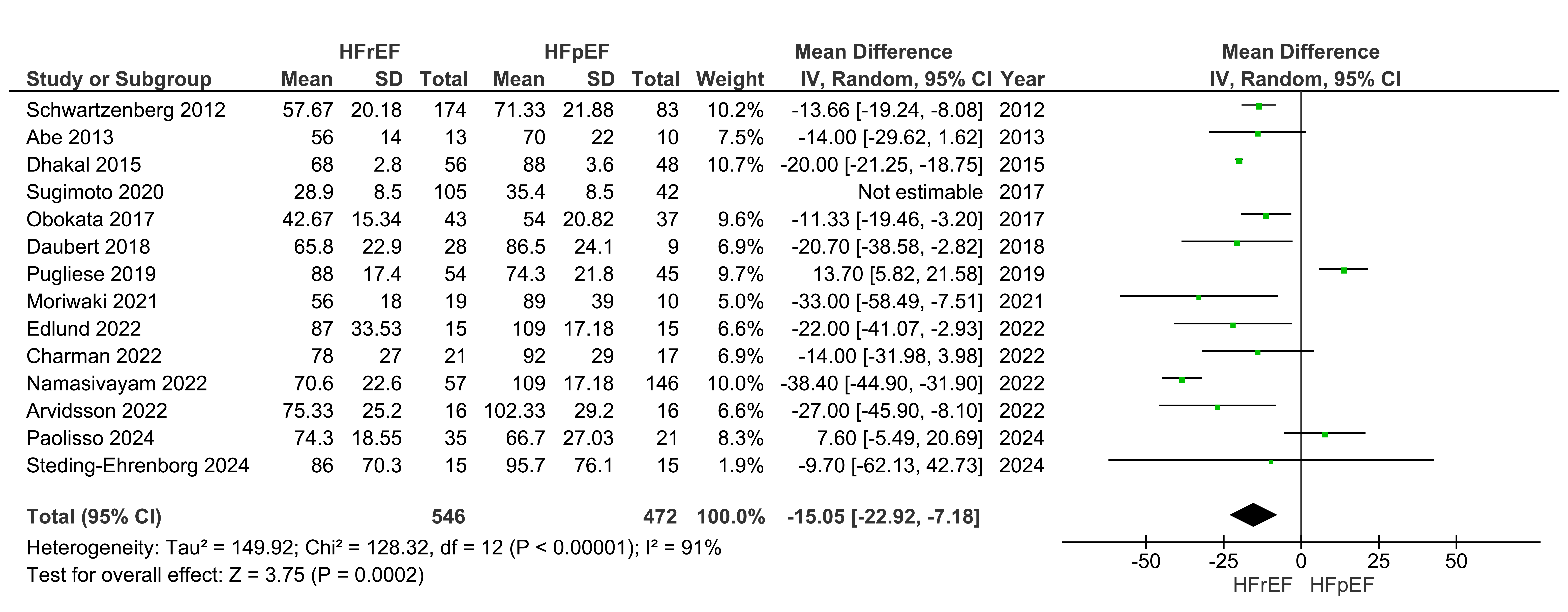

Supplement: oeaf055_Supplementary_Data [file oeaf055_supplementary_data.zip › Figure S5.tiff]

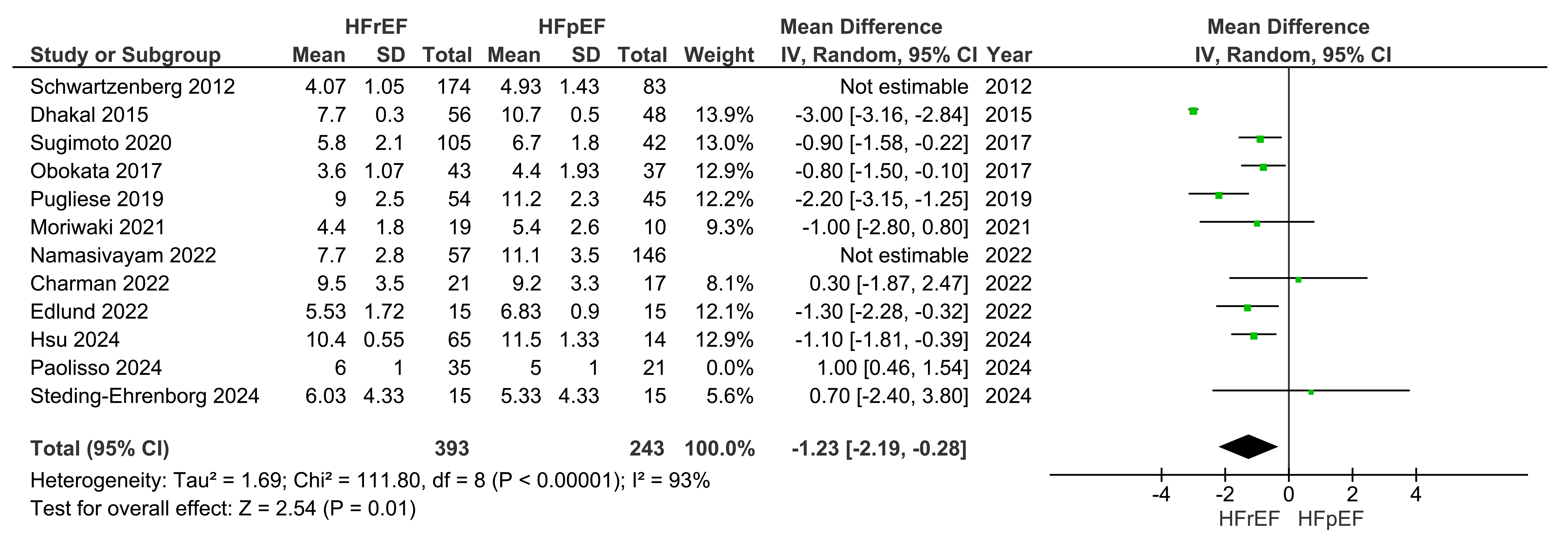

Supplement: oeaf055_Supplementary_Data [file oeaf055_supplementary_data.zip › Figure S6.tiff]

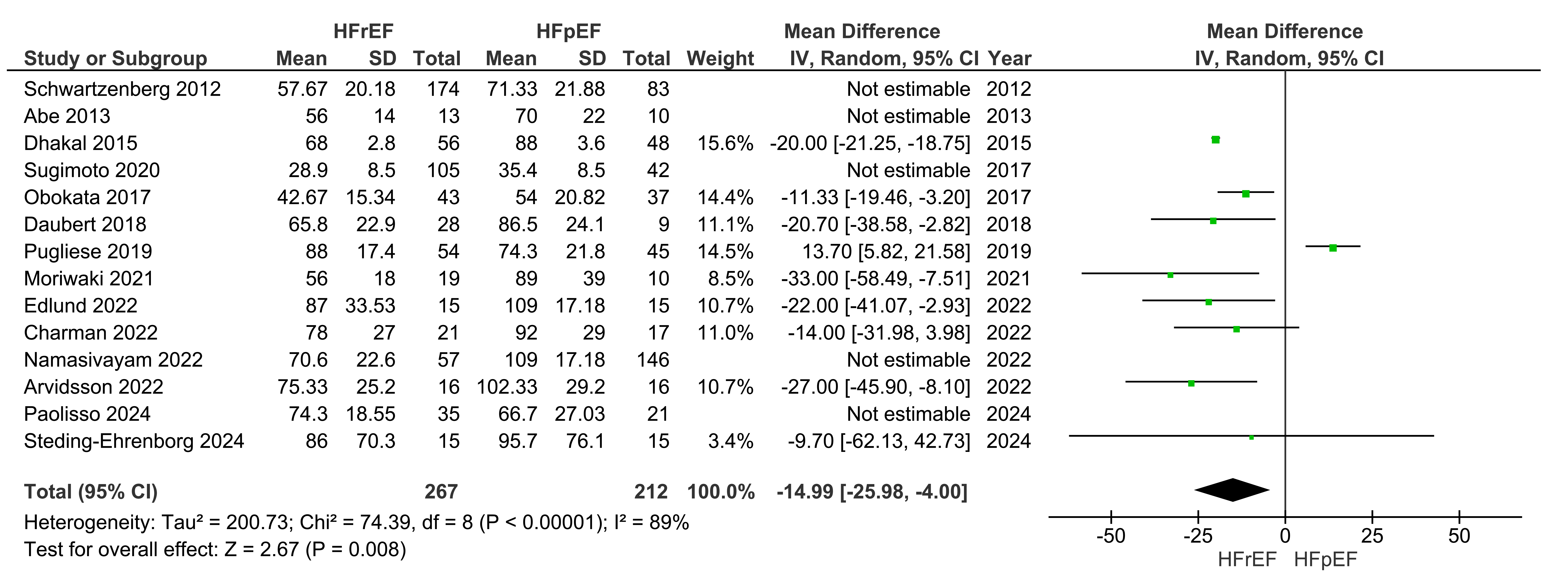

Supplement: oeaf055_Supplementary_Data [file oeaf055_supplementary_data.zip › Figure S7.tiff]
